# Operando monitoring of dendrite formation in lithium metal batteries via ultrasensitive tilted fiber Bragg grating sensors

**DOI:** 10.1038/s41377-023-01346-5

**Published:** 2024-01-22

**Authors:** Xile Han, Hai Zhong, Kaiwei Li, Xiaobin Xue, Wen Wu, Nan Hu, Xihong Lu, Jiaqiang Huang, Gaozhi Xiao, Yaohua Mai, Tuan Guo

**Affiliations:** 1 Institute of Photonics Technology, Jinan University, Guangzhou, 510632 China; 2 Institute of New Energy Technology, College of Information Science and Technology, Jinan University, Guangzhou, 510632 China; 3 Key Laboratory of Bionic Engineering (Ministry of Education), Jilin University, Changchun, 130022 China; 4 The Key Lab of Low-Carbon Chemistry & Energy Conservation of Guangdong Province, School of Chemistry, Sun Yat-Sen University, Guangzhou, 510275 China; 5 Sustainable Energy and Environment Thrust, The Hong Kong University of Science and Technology (Guangzhou), Nansha, Guangzhou, Guangdong 511400 China; 6 Advanced Electronics and Photonics Research Centre, National Research Council of Canada, Ottawa, K1A 0R6 Canada

**Keywords:** Optical sensors, Imaging and sensing, Fibre optics and optical communications

## Abstract

Lithium (Li) dendrite growth significantly deteriorates the performance and shortens the operation life of lithium metal batteries. Capturing the intricate dynamics of surface localized and rapid mass transport at the electrolyte–electrode interface of lithium metal is essential for the understanding of the dendrite growth process, and the evaluation of the solutions mitigating the dendrite growth issue. Here we demonstrate an approach based on an ultrasensitive tilted fiber Bragg grating (TFBG) sensor which is inserted close to the electrode surface in a working lithium metal battery, without disturbing its operation. Thanks to the superfine optical resonances of the TFBG, in situ and rapid monitoring of mass transport kinetics and lithium dendrite growth at the nanoscale interface of lithium anodes have been achieved. Reliable correlations between the performance of different natural/artificial solid electrolyte interphases (SEIs) and the time-resolved optical responses have been observed and quantified, enabling us to link the nanoscale ion and SEI behavior with the macroscopic battery performance. This new *operando* tool will provide additional capabilities for parametrization of the batteries’ electrochemistry and help identify the optimal interphases of lithium metal batteries to enhance battery performance and its safety.

## Abstract (web summary)

Operando monitoring of mass transport kinetics and lithium dendrite growth in lithium metal batteries and parametrization of the batteries’ electrochemistry and safety have been achieved using optical fiber sensors.

## Graphical abstract

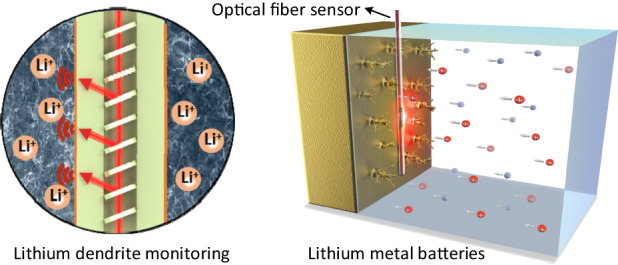

## Introduction

The extensive reliance on traditional fossil energy sources like petroleum and coal has engendered significant challenges in contemporary energy systems, manifesting as energy shortages and environmental pollution. As society moves towards carbon neutrality, a crucial piece of the puzzle for harvesting renewable energies is to develop advanced energy storage systems, such as batteries and supercapacitors, that can store and deliver significant amounts of charge and that can withstand many thousands of charge–discharge cycles^1–4^. Conventional lithium-ion batteries (LIBs) can barely break through their theoretical energy density limits. Therefore, it is particularly important to develop novel battery systems that go beyond conventional LIBs^5^. Rechargeable lithium metal batteries (LMBs) are considered as the “Holy Grail” of energy storage systems, because of their ultrahigh theoretical specific capacity (3860 mAh g^−1^) together with the lowest negative electrochemical potential (−3.04 V versus the standard hydrogen electrode)^6–8^. Despite these exceptional merits, the uncontrolled lithium dendrites growth during extended cycling gives rise to capacity fading, short circuits, and potentially catastrophic failure^9–11^. Consequently, the growth of lithium dendrites remains one of the most serious challenges for the implementation of next-generation, high-energy-density LMBs.

The formation of detrimental lithium dendrites is dependent upon a few key factors^12,13^, such as mass transport and solid electrolyte interphase. Learning from the electrodeposition of copper, the importance of mass transport on dendrite formation is immediately recognized^14^. In particular, the morphology of deposited lithium transitions from mossy to dendritic, once the lithium ions have been depleted in the electrolyte near the lithium metal electrode^15,16^. Another mechanism of dendrite formation stems from the unregulated reactions on the active lithium surface to form SEIs, thereby creating spatial heterogeneities. Intrinsically, the heterogeneous SEI induces inhomogeneous mass transport or even retards the lithium diffusion, offering nucleation sites for dendrite formation. The dendrite exposes more fresh lithium metal to the organic electrolyte, continuously generating new SEIs and dendrites. The proliferating dendrite growth consumes both active lithium and electrolyte, leading to capacity degradation, resistance buildup, and electrolyte depletion. Thus, it is necessary to regulate the mass transport in electrolytes and to control the SEI formation^13^. Creating artificial SEIs with optimal mechanical and transport properties turns out to be an effective strategy for stabilizing lithium metal anodes. However, the relationship between the artificial SEIs and the mass transport in the electrolyte at the interface remains poorly understood.

One formidable obstacle to unfolding the interrelation is the difficulty of measuring the temporally and spatially dynamic mass transport at the electrolyte–electrode interface. That difficulty originates mainly from the strong “gap” at the such interface, i.e., the relatively low concentration (<4 M) and high diffusion coefficient (ca. 10^−^^6^ cm^2^ s^−1^) of the electrolyte, in comparison to those of solid-phase active materials^15^ (10–50 M; <10^−9^ cm^2^ s^−1^). These differences make monitoring the mass transport at the interface much more difficult. More stringent measurements are required with ultrahigh sensitivity and superfine temporal and spatial resolution. Pioneering progress has been made to track the electrolyte concentration gradients. For example, Grey et al. applied magnetic resonance imaging^16^ to measure the electrolyte concentration gradients and correlated them to the lithium metal microstructure. However, their measurements came at the expense of a relatively long acquisition time (tens of minutes). More recently, stimulated Raman scattering microscopy and spontaneous Raman scattering microscopy^17^ have been proposed for visualizing of the concentration profiles of bis (oxalato) borate anions with a much-reduced acquisition time of tens of seconds. These measurements revealed the interplay between the local ionic concentration and lithium dendrite growth. However, all these sophisticated characterization techniques are not capable of monitoring commercial batteries under real working conditions and at end-user applications. The above review highlights the urgent need for easy-to-use and practical sensing technologies that can be seamlessly integrated into a battery pack for continuous monitoring the mass transport of a battery’s electrolyte during the lifelong operation.

In recent years, optical fiber sensors have presented a high degree of miniaturization and a relatively simple-to-implement for a variety of hard-to-access environments^18^. Optical fiber sensors, with a diameter less than 0.2 mm, can be embedded into batteries near the surface of the battery electrodes, ensuring minimal disruption to battery operation. They offer high sensitivities and fast responses to the various key parameters of the batteries (temperature, pressure, strain, refractive index of the electrolyte and gases, and their compositions) by interrogating the light’s wavelength, intensity and polarization^19–22^. The initial study of real-time temperature measurements of batteries using optical fiber sensors, was pioneered by Pinto’s group^23^. Subsequently, Tarascon’s group successfully tracked the solid electrolyte interphase (SEI) formation and structural evolution by simultaneously measuring the internal temperature and pressure of commercial cell using a fiber Bragg grating (FBG) and a micro-structured optical fiber^24^. And Huang’s team employed FBG sensors implanted into lithium–sulfur cells for accurate estimation of the cathode stress and its evolution. The results indicate a close correlation between stress evolution and the characteristics of cathode structure and volume changes^25^. More recently, significant progresses have been made in the chemical and electrochemical optical fiber sensing methods, by using TFBG sensor^26–28^, hollow-core fiber sensor, and infrared optical fiber evanescent wave spectroscopy^29^. These methods enable in situ monitoring of electrolyte–electrode chemistry, which significantly enhanced the comprehension of battery state of charge, electrolyte composition and aging and safety risk assessment. For example, Euser’s group embedded a novel hollow-core optical fiber probe inside a battery, and monitored the evolution of electrolyte species by Raman spectroscopy to reveal changes in the lithium-ion solvation dynamics^30^. In summary, researchers are extensively engaged in the pursuit of advanced tools capable of measuring physical, chemical, and electrochemical parameters with high temporal and spatial resolution within batteries and during their operation. This concerted effort aims to enhance our comprehension of the fundamental electrochemical processes. Simultaneously, it holds the potential to yield practical systems deployable for monitoring installed systems and providing valuable insights for maintenance and replacement schedules.

In this paper we demonstrate an ultrasensitive superfine-resonance optical fiber sensor inserted into batteries for in situ and continuous monitoring of mass transports in electrolytes near the electrolyte–electrode interface of rechargeable lithium metal batteries, without perturbing battery operation. The sensor provides a scalable solution for tracking the mass transport kinetics affected by SEI formation, and monitoring lithium dendrite growth on different Li anodes variants. Further analysis reveals stable and reproducible correlations between cells’ performance and the time-resolved optical responses, allowing parametrization and quantification of the battery’s electrochemistry. This new *operando* measurement tool introduces a crucial additional functionality for battery monitoring methods, offering comprehensive guidance for the design of better batteries with improved safety and electrochemistry.

## Results

### Concept and methodology

**Fig. 1:**
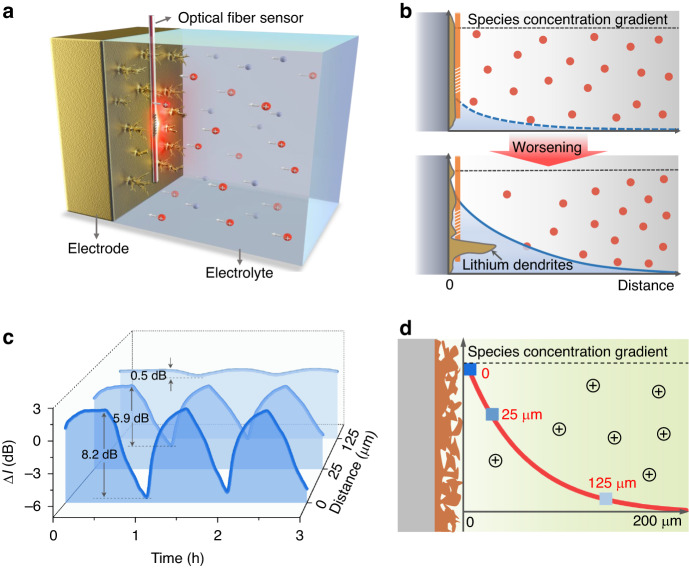
The concept of optical fiber in situ detection at the lithium electrode interface. **a** Schematic diagram of a TFBG sensor positioned in close proximity to the electrolyte–electrode interface of a lithium metal battery. This configuration enables in situ detection of interfacial species concentration and mass transport. **b** Schematic plots for species concentration distribution (blue curves), and corresponding lithium dendrites morphologies. **c** Spatial-temporal evolution characteristics of species concentration near the surface of the lithium anode as verified by an optical fiber sensor. The distance between optical fiber sensor and lithium surface is 0, 25, and 125 μm, respectively. **d** Spatial distribution of species concentration (red fitting line) near a lithium anode during lithium plating

As mentioned, mass transport in electrolytes largely governs the electrochemical plating and stripping of lithium metal anodes but remains challenging to monitor. To *operando* track the mass transport kinetics, we imprinted a tilted fiber Bragg grating in the core of a commercial single-mode fiber (Fig. S1). We then implanted the TFBG sensing probe into the battery and tightly attached it to the electrode’s surface while a fixing device (Fig. 1a and Fig. S2). Due to the inherent light coupling properties of TFBGs^31^, part of the incident optical power is coupled from the fiber’s core into the cladding, so that its evanescent field extends beyond the fiber’s surface and penetrates surrounding media (e.g., the electrolyte and the electrode surface), as shown in the simulation results in Fig. S1. The strong interaction between the evanescent field of the TFBG and the surrounding electrolyte offers the capability to *operando* monitor the electrolyte kinetics with high sensitivity^32^.

The difference in mass transport kinetics leads to different species concentration gradient distribution near the electrode, which induces the formation of lithium dendrites with different morphology^12^ (Fig. 1b). In details, a gently sloping concentration gradient (the blue dotted line) indicates healthy mass transportation kinetics adjacent to the electrode. On the other hand, a steeper concentration gradient (the blue line) indicates worsened mass transportation kinetics which may relate to the growth of lithium dendrites. Therefore, it is important to in situ measure the species concentration gradients versus distance from the electrode surface, to reveal the spatiotemporal evolution characteristics of the species concentration. As shown in Fig. 1c, the optical signal response intensity of optical fiber sensor caused by species concentration changes gradually reduces from a strong modulation (8.2 dB at electrode surface) to a much lower level (0.5 dB far from the electrode surface), see more details in Fig. S3. And the Fig. 1d indicates that the species concentration at the electrode surface is much lower than that in the bulk electrolyte during lithium electrodeposition, which is limited by the mass transfer rate during the electrochemical reaction.

**Fig. 2:**
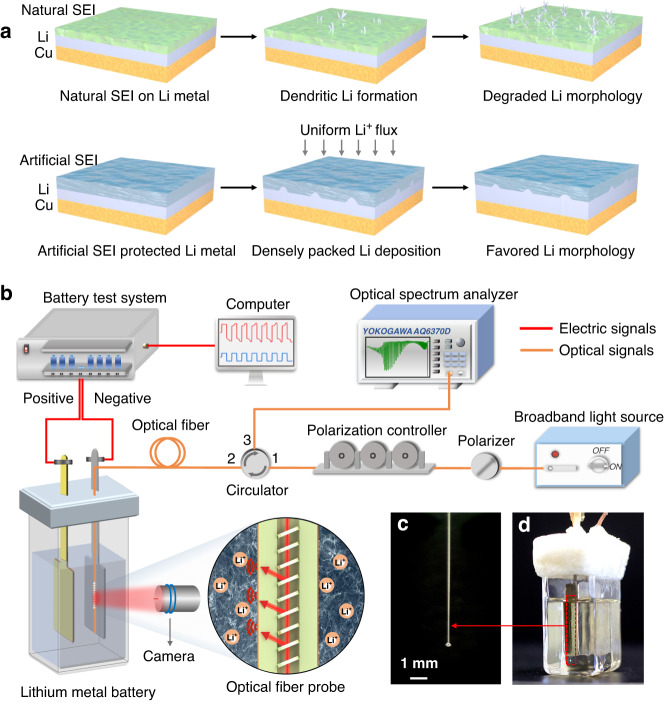
Schematic illustration of the lithium plating behavior of natural and artificial SEI Li anode structures and sensing system for operando monitoring of a lithium metal battery. **a** Scheme of the lithium metal morphological evolution with cycling for a natural SEI (top) and artificial SEI (bottom). **b** Experimental setup of an evanescent optical fiber sensing system for monitoring the ionic concentration of lithium metal battery at electrolyte–electrode interface. The zoomed inset presents the idea of capturing the localized ionic concentrations and fast ion transport kinetics over the lithium anode interface using an implanted optical fiber sensor. **c**, **d** Photographs of the optical fiber sensing probe and lithium metal anode symmetrical cell, respectively

The study of lithium plating process encompasses a series of intricate chemical and electrochemical processes^33^, and most of these processes are intricately connected to the interplay between the mass transport in the electrolyte and SEI layer^34,35^. In recent years, much more attention has been concentrated on the artificial SEI study, owing to its effectiveness in enhancing performance of battery and its good potential for scalable manufacturing. In comparison to an unprotected lithium anode, the artificial SEI promotes an even distribution of lithium-ion flux across the electrode surface and a more uniform lithium plating/stripping behavior (Fig. 2a). In order to unravel the relationship between the mass transport kinetics of electrolyte and lithium plating, we constructed an all-fiber-coupled electrochemical optical fiber sensing system (detailed information refer to the Method section), consisting of a TFBG probe and associated optical components, and deployed it on a purpose-constructed symmetrical Li metal anode battery, as shown in Fig. 2b. The optical fiber sensor was securely affixed to the surface of the lithium metal anode and acted as a fixed device (see the microscope images in Fig. S2). This configuration, with the cut fiber end free eliminates the effects of cross-sensitivity to strain and polarization. Meanwhile, a long focal length camera with a high spatial resolution (finer than 1 μm) was used for simultaneously imaging the morphology of lithium dendrite growth close to the fiber sensor. The TFBG-imprinted optical fiber probe is with a length of 18 mm and a diameter of 125 μm (see Fig. 2c). And the whole photograph of the lithium metal battery with integrated optical fiber sensing probe is shown in Fig. 2d.

**Fig. 3:**
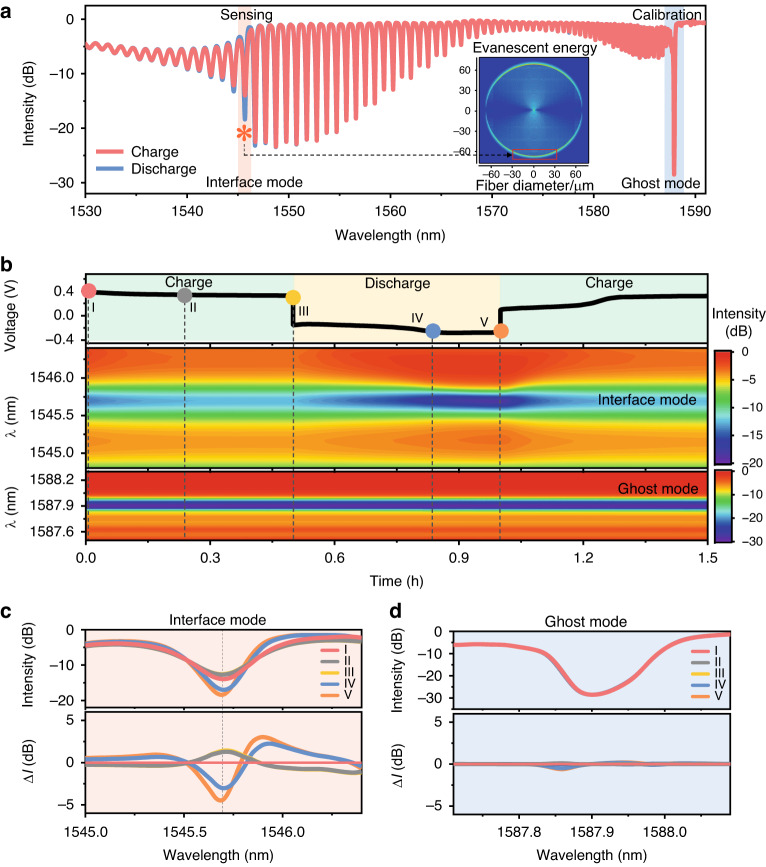
Spectral response of optical fiber sensor. **a** Spectral response of the TFBG optical sensor (with a tilt angle of 16°) versus cell charging and discharging, in which the most sensitive interface mode (at the wavelength of 1546 nm, marked with an orange asterisk “*”) is used for species concentration monitoring, and the ghost mode resonance at 1587 nm is used to track and compensate for temperature fluctuations. The inset shows a simulated optical field distribution along the fiber sensor’s cross-section. **b** Temporal voltage (top trace), spectral responses of interface mode resonance (middle) and ghost mode resonance (bottom) of the optical fiber sensor during the first charging cycle of a lithium metal battery. **c**, **d** Original (up) and differential (down) spectral responses of the interface mode (changed) and the ghost mode (almost unchanged)

Figure 3a shows typical comb-like reflection spectra from a TFBG during the charge–discharge cycling of a lithium metal battery. Due to the introduction of the tilt angle of the grating, the forward-propagating incident light of the TFBG is effectively coupled into the backward-propagating cladding mode, and the backward-propagating core mode that satisfies the Bragg condition is retained^31,36^. The resonant peaks of tens of narrowband cladding modes, each with a full width at half maximum (FWHM) of about 0.2 nm, are evident at shorter wavelengths based on the core-to-cladding resonant mechanism. These cladding mode resonances are extremely sensitive to variations in the refractive index of the surrounding medium. Notably, TFBGs operating in the near-infrared spectrum offer a significantly longer penetration depth (1.5 μm), which encompasses the most active regions for electron transfer and ion transport across the electrode surface. Any subtle alterations in ion distribution around the electrode can be directly monitored by observing the changes in the amplitude of the cladding resonance. Meanwhile, the high-amplitude dip at 1587 nm (blue shading, Fig. 3a), the so-called “ghost” mode, arises from guiding of core diffracted light along the core–cladding interface. The ghost mode is immune to the surrounding media and can be used for temperature measurement.

We designate the cladding mode which shows the greatest sensitivity to surrounding refractive index (SRI) as the “interface mode” (identified by an orange asterisk * in Fig. 3a). The interface mode is able to track perturbations in the ambient refractive index with higher sensitivity than any cladding mode resonance (comprehensively analyzed in Fig. S4). Specifically, the resonance amplitudes of the interface mode are suppressed by decreasing the LiPF_6_ concentration in the electrolyte, corresponding to a refractive index increase (see full analysis in Fig. S5). Figure 3b shows the voltage profile (top) and the spectral evolution of a TFBG’s interface mode resonance and Bragg resonance (middle and bottom traces, respectively) during the first charge–discharge cycle of the lithium metal battery at a current density of 1 mA cm^−2^. The amplitude of the resonance at 1545.6 nm gradually decreases during charging and reaches a maximum near the end of discharging. We note that the amplitude and wavelength of the ghost mode remain unchanged throughout the charge–discharge cycle (Fig. 3b, bottom). To further aid visualization of the spectral evolution, we recorded the interface and core mode spectra at five times during the cycle (colored dots in Fig. 3b) and presented their spectra in Fig. 3c, d, top. We quantified the intensity change (Δ*I* = *I* − *I*_ini_) of the interface mode and ghost mode relative to that of initial states (*I*_ini_) in Fig. 3c, d, bottom. We see that the intensity of the ghost mode remains almost unchanged (Fig. 3d), while the intensity of the interface mode varies considerably throughout the electrochemical cycle (Fig. 3c). The interface mode changes reflect the cycling of electrolyte composition in the proximity of the lithium/electrolyte interface during charging and discharging.

### Probing mass transport in electrolyte via TFBG

**Fig. 4:**
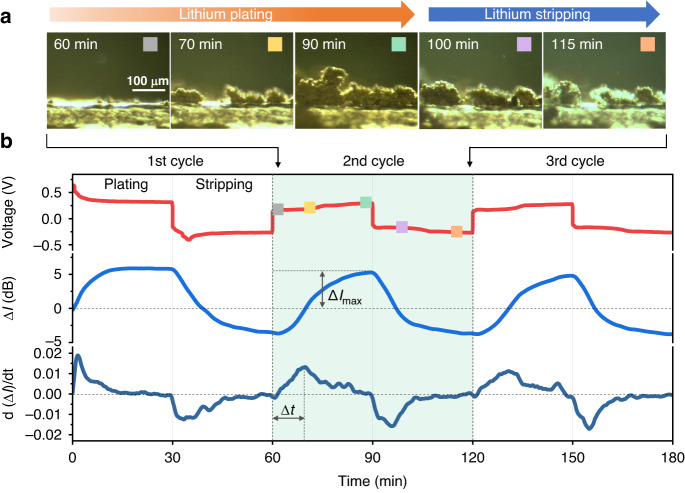
Tracking of species concentration at electrode interface and analysis of the kinetic process. **a** Dark field optical microscope images of lithium dendrites during 2nd cycling, clearly revealing the lithium plating and striping on the surface of the bare lithium anode. **b** Periodic voltage variation with time (top), the corresponding periodic variation of interface mode amplitude of the implanted TFBG (middle), and its differential optical power evolution (bottom) in charge–discharge cycling tests of the lithium battery. It defines the maximum optical power change (∆*I*_max_), and the response time (∆*t*) achieved to the peak of the differential of optical power in the lithium plating process

A high-concentration electrolyte (4 M LiPF_6_ in a 1:1:1 mixture of ethylene carbonate (EC), ethyl methyl carbonate (EMC) and dimethyl carbonate (DMC)) was used for reducing the lithium diffusion coefficient^37^, thereby smoothing the average concentration gradient near the electrode surface. It is noteworthy that electrolytes with ultrahigh salt concentration can effectively shield the lithium metal anode from the formation of lithium dendrites, thereby creating a battery that exhibits outstanding electrochemical performance and high safety^38,39^. To better understanding the correlation between the dynamic process of lithium plating/stripping and the optical spectral response, we recorded the microscope images of lithium dendrites over the second cycle of charging and discharging, as shown in Fig. 4a. Images during discharging at times 100 and 115 min, showing that the lithium dendrites gradually shrink, clearly revealing the lithium stripping during the discharge process. The dynamic evolution of the lithium plating/stripping process is presented in the Supplementary Movie. Voltage profiles of the lithium/lithium symmetrical cell together with the corresponding optical sensor signals during the galvanostatic charge–discharge are shown in Fig. 4b. The voltage polarizations of the cell are slightly reduced right upon the first plating (Fig. 4b, top), due possibly to the activated process with SEI formation of lithium plating/stripping^40,41^. To simplify the analysis, we present Δ*I* rather than the raw spectra of the interface mode in Fig. 4b. Clearly, the optical signal response (Δ*I*, depicted in Fig. 4b as the blue curve) obtained from the TFBG sensor exhibits a strong correlation with the voltage signal (Fig. 4b, red curve).

∆*I* represents the real-time difference of optical sensor signal between the instantaneous and initial values, which we interpret as the refractive index changes within the Nernst diffusion layer of liquid electrolyte, and consequently, reflects the mass transport within the electrolyte. Mass transport could result from electromigration, gradient-driven diffusion, and advection. In the ideal case, during the initial lithium plating, the metal cations in the electrolyte first migrate under the influence of the electric field and then form a concentration gradient of species with lower species concentrations near the deposition electrode^12^. This could explain why ∆*I* increases with time, corresponding to lower species concentrations than were observed in the bulk electrolyte (Fig. S5) during the first lithium plating (Fig. 4b). Dyring the subsequent lithium stripping process, ∆*I* decreases (Fig. 4b), which is explained by the reversed lithium-ion flux in the electrolyte.

In practice, the irreversible SEI-related electrolyte decomposition on active lithium can also contribute to the variation of ∆*I*. As Newman et al. stated^42^, in such an electrochemical system, reactions, including the electrolyte decomposition, are frequently restricted to the electrode surface. However, the sensing point at the Nernst diffusion layer could still discern these reactions indirectly through mass transport. Notably, the ∆*I* profile shows a periodic pattern after the first plating (Fig. 4b). The absence of a visible shift in the baseline background in the first three cycles (Fig. 4b) implies that the bulk electrolyte remains almost intact even in the presence of the electrolyte decomposition, owing possibly to the excess electrolyte in the battery. However, we do observe a minor change in ∆*I* background after 12 cycles (Fig. S6), indicating a noticeable degradation of bulk electrolyte. In brief, the ∆*I* variations stem from the electrochemical lithium plating/stripping and the accompanying SEI-related electrolyte decomposition, both of which influence the mass transport in the Nernst diffusion layer.

To extract more information from the optical signal, we further took the derivative of ∆*I* with respect to time. The rationale behind this could trace back to the transport equation^42^ in the differential form without the mass production term,

$$\frac{\partial {c}_{i}}{\partial t}=-\nabla \cdot {J}_{i}$$

where ${c}_{i}$ is the concentration of species *i*, $t$ is time, $\nabla {\rm{\cdot }}$ is divergence, and ${J}_{i}$ is the flux of species *i*. Since the ∆*I* is tightly linked to the concentration (${c}_{i}$) change as seen in Fig. S5, the d(∆*I*)/d*t* could imply the divergence of mass flux ($-\nabla \cdot {J}_{i}$, or $-\frac{\partial {J}_{i}}{\partial x}$ in one-dimensional form) and provide more relevant information about mass transport (Fig. S7). The mass transfer rate rapidly increases at the beginning and then gradually tends to level off, which implies the mass transfer undergoes an unsteady-state migration process and then gradually approaches a steady state during lithium plating^43^. Therefore, the response time (∆*t*) achieved at the peak of the differential of optical power can be used to characterize the state of mass transport. Shorter Δ*t* indicates faster mass transfer in the Nernst diffusion layer, enabling rapid replenishment of ion depletion at the electrode interface to suppress the rate of decrease in species concentration. In short, a faster mass transfer rate can effectively reduce the species concentration gradient at the electrode interface. Therefore, homogeneous mass distributions on the surface of lithium metal anode are beneficial for lithium dendrite growth suppression.

### Characterization anodes with different artificial interfaces

**Fig. 5:**
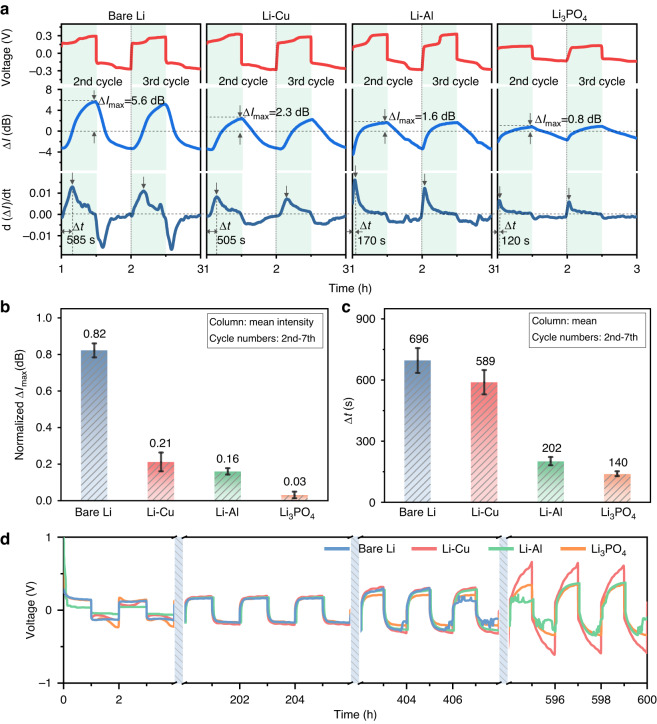
Analysis of the regulation of lithium plating by the SEI layer. **a** Temporal voltage (top), the interface mode amplitude variation of TFBG (middle), and the evolution of differential optical power d(∆*I*)/d*t* (bottom) during the stable cycling of lithium batteries with modified SEI layers. Similar to Fig. 4, ∆*I*_max_ in Fig. 5 reveals the maximum variation in species concentration at the lithium anode interface during the lithium plating stage. In the bottom panel, ∆*t* reflects the response time achieved to the peak of the differential of optical power during lithium plating. **b**, **c** Quantitative comparison of mass transport at different modified interfaces during lithium plating. **d** Cycling comparison for Li/Li, Li–Cu/Li–Cu, Li–Al/Li–Al, and Li–Li_3_PO_4_/Li–Li_3_PO_4_ symmetric cells at a current density of 0.5 mA cm^−2^

As previously mentioned, the complex electrochemical process involving mass transport and the interaction of the SEI layer is involved in the lithium plating process. However, how the SEI regulates Li dendrite growth and how to design a better SEI remain unclear. Therefore, we used three types of conductors, namely ionic conductor, electronic-ionic mixed conductor, and electronic conductor, to construct three artificial protection layers. The layer compositions we investigated were Li_3_PO_4_^9^, Li_*x*_Al alloy^44^ and Cu nanoparticles^45^. We then implanted a TFBG optic sensor into each battery for continuous, in situ monitoring of the interfacial mass transport kinetics within the Nernst diffusion layer of lithium metal anode surfaces. We note that the formation of the SEI layer during the initial cycling process may lead to complex reaction and mass transfer at surface of the bare lithium anode. Therefore, here we focused on the following cycling processes for comparison. The traces in Fig. 5a present the relationship between electrochemical signal, optical signal, and derivative of ∆*I* with respect to time for three anodes with the different artificial protection layers during, as well as with bare Li for comparison. Therefore, by constructing an artificial protection layer on the lithium metal anode surface, we can effectively decrease the values of ∆*I*_max_ and ∆*t* relative to the bare Li anode.

To eliminate potential differences in spectral amplitude response attributable to electrochemical parameters, we initially normalized the ∆*I*_max_ values based on the maximum optical power variation observed in bare Li during the 2nd to 7th cycles, and then performed statistical analysis on the normalized results to obtain the bar graph shown in Fig. 5b. We found that the normalized ∆*I*_max_ values corresponding to Li–Cu and Li–Al anode are reduced by factors of 0.61 and 0.66, respectively, compared with the bare Li anode. For the Li–Li_3_PO_4_ anode, the normalized ∆*I*_max_ values drop significantly, declining by a factor of 0.79. Apparently, the bare Li anode surface exhibits the highest concentration gradient, which further exacerbates Li dendrite formation in the bulk electrolyte. As mentioned above, the SEI layer greatly influences the final morphologies of electroplated metals. During the lithium plating process, the SEI layer with high ionic conductivity will accelerate ion diffusion toward the metal surface, thereby establishing a low concentration gradient^36^. The bar graph in Fig. 5c shows the statistical results of ∆*t* of different electrodes. As expected, the enhanced effect of the Li_3_PO_4_ SEI layer on the mass transport at the lithium anode interface increases the mass transfer rate of species in the electrolyte, which in turn exhibits a lower ∆*t* (Fig. 5c).

We investigated the many-cycle stability of symmetrical coin cells without TFBG to further demonstrate the relationship between mass transport behavior and conductive mechanisms of artificial SEI layer. The symmetrical coin cells with four kinds of lithium anodes were assembled with 4 M LiPF_6_ EC: EMC: DMC electrolyte, and then each was cycled at 0.5 mA cm^−2^ with areal capacity of 0.5 mAh cm^−^^2^. Figure S8a shows the symmetrical cell with Li–Li_3_PO_4_ anode delivers the best long-term cyclic performance together with a minimized voltage polarization beyond 600 h. The voltage profiles of the cells at selected cycle ranges are provided in Fig. 5d. Variances are evident at 406–408 and 594–596 h, signifying that the cell with Li–Li_3_PO_4_ anode exhibited a normal lithium plating/striping voltage, the cell with Li–Cu anode demonstrated a significant increase in voltage polarization, and the cells with bare Li and Li–Al anode are likely experiencing short-circuited. The morphologies of deposited lithium metal anodes before and after 50 cycles were characterized by scanning electron microscopy (SEM) to further confirm the effect of the artificial SEI layers on the lithium plating process (Fig. S9). The bare Li anode showed a typical morphology of lithium dendrite growth just after the 50th cycle. In comparison, the Li–Li_3_PO_4_ anode and Li–Al anode maintained a smooth surface without dendrites. These observations suggested that the Li_3_PO_4_ and Li_x_Al layers can greatly suppress dendrite growth. Interestingly, the porous surface of Li–Cu anode became smooth after cycling, possibly caused by the electrolyte decomposition compounds filling in the pores. That is further confirmed by energy dispersive spectroscopy (EDS) (Fig. S10) and ex situ X-ray photoelectron spectroscopy (XPS) (Fig. S11), which exhibits the highest LiF ratio in the fluorine 1s region spectrum of the Li–Cu anode. These characterization results exhibit correlated consistency with spectral changes. In particular, the in situ monitoring of the mass transport kinetics at the electrode interface by the TFBG sensor can reflect the regulatory behavior of the artificial SEI layer on lithium plating.

### Quantification of mass transport kinetics for different SEI layers

**Fig. 6:**
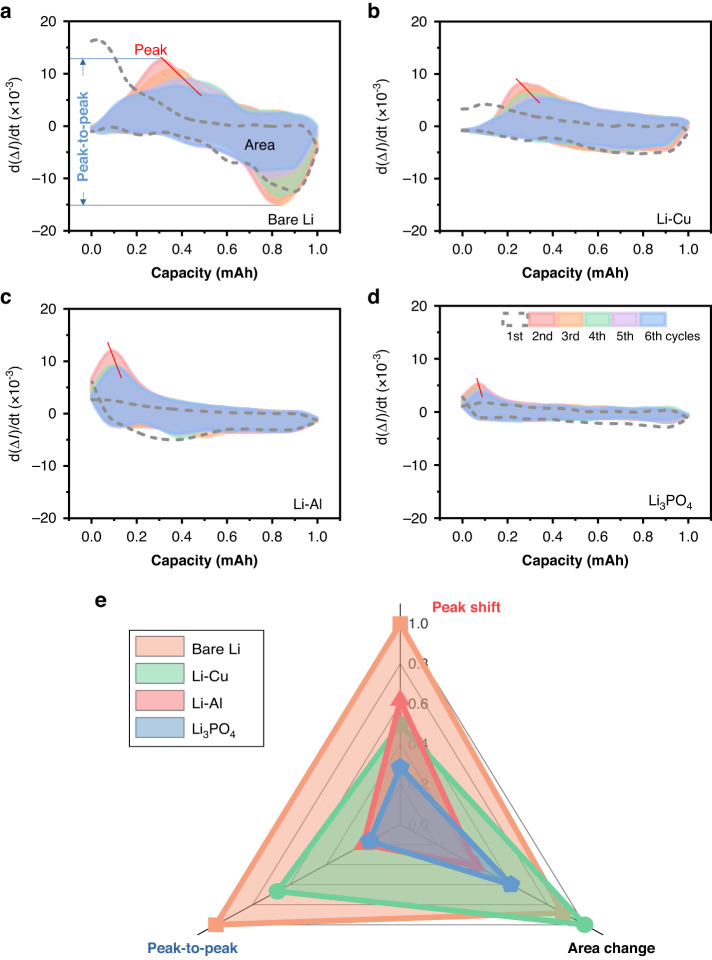
Quantification of the mass transport kinetics for different SEI layers. **a-d** Establishing a correlation between the evolution of the differential interface mode amplitude (d(∆*I*)/d*t*) and the battery capacity. The red dotted line indicates a shift of the peak of differential optical power in charge–discharge cycling tests of lithium metal batteries. **e** Quantitative comparison of the normalized area change, peak-to-peak value, and peak drift of the ∆*I*′/*Q* curve in (**a**–**d**). The change in area is defined as the difference between the maximum and minimum areas divided by the maximum area

As both the optical and electrochemical signals are time-dependent, the rate of spectral amplitude change (d(dB)/dt, denoted as ∆*I*′) can be correlated to the capacity (*Q*) through a ∆*I*′/*Q* relationship curve. This mapping curve unambiguously illustrates the rate of change in ionic concentration at the electrolyte–electrode interface, which provides a precise quantitative and easy understanding approach for the study of ion kinetics and electrode interactions. Figure 6a–d present the ∆*I*′/*Q* mapping curves of the first six lithium plating/stripping cycles, which indicate the ionic concentration rate of change with capacity, for three different SEI layer compositions and bare Li. The first cycle is partly irreversible due to the formation of the SEI layer. Crucially, we observe a consistently reproducible correlation between battery capacity and the rate of species concentration change. Compared to the bare Li symmetric cell, the area covered by the ∆*I*′/*Q* mapping curve corresponding to the Li_3_PO_4_ symmetric cell varies more gently. This is attributed to the suppression of side reactions between the lithium anode and the electrolyte due to the existence of a dense and robust of the Li_3_PO_4_ layer for stable electrochemical performance.

Finally, Fig. 6e presents a synthesized radar chart for the evaluation of battery’s mass transport characteristics and the chemical stability of the SEI layer. It includes the three parameters: peak-to-peak value, peak shift, and area change of the ∆*I*′/*Q* mapping curve; The smaller the size of the triangle, the better the battery performance. It can be clearly observed that the Li_3_PO_4_ electrode (the blue one) exhibits the best electrochemical performance, both in terms of lithium dendrite inhibition and cycle stability. This is consistent with the conclusion mentioned above that the ionic conductor SEI layer has apparent advantages in regulating the uniformity of ion species concentration. This result further confirmed that the in situ optical fiber sensing system can be used for real-time monitoring of mass transport in lithium battery electrolytes. Moreover, the *operando* monitoring capability of optical fiber sensors offers further insights into key parameters for designing an effective SEI layer.

## Discussion

We have demonstrated that the TFBG sensors can be used for *operando* and continuous monitoring of the mass transportation kinetics in Nernst diffusion layer of lithium metal anode surfaces under real working conditions. These sensors provide a more simplified and reproducible method to measure refractive index of electrolyte. Moreover, we provide evidence that the all-fiber-coupled electrochemical sensing system can precisely measure the mass transfer in the proximity of an electrode surface during battery operation. Based on the rationally designed artificial SEI layers with different types of transport mechanisms (such as ionic conductor, electron-ion mixed conductor, and electronic conductor) on a lithium metal electrode, and then combined with the optical fiber sensors *operando* monitoring method, the obtained results reveal an intrinsic correlation between the mass transfer and dynamic lithium dendrite growth. We have confirmed that the artificial SEI layer based on an ionic conductor most effectively promotes rapid convergence of mass transport to a steady state, which is beneficial for suppressing lithium dendrite growth. The as-developed *operando* monitoring method can shed fresh light into the design principles of the of long-lifespan, high-energy-density, and high-security lithium metal batteries.

With society’s extensive dependence on rechargeable batteries, especially for electric vehicles, the development of a portable and cost-effective interrogator for in-field measurements becomes crucial. A feasible way is to use a compact tunable laser (like VCSEL) and a photodiode, together with an analog-to-digital converter (A/D) for data processing, see Fig. S14. Such laser may offer a fixed or a narrow band tunable light, with wavelength match to the most sensitive resonance of the sensor. However, temperature compensation and power fluctuation suppression should be considered in this way.

Overall, truly multidisciplinary efforts between battery science, photonics, and data science opens up unprecedented opportunities for battery diagnostics (Fig. S15). Based on the multiple mode response of TFBG to different physical quantities, a “lab-on-fiber” analysis platform can be established to obtain and decouple multiple parameters inside the battery at the same time. Additionally, this *operando* techniques can be extended to other energy storage devices (such as supercapacitors and fuel cells) and other important applications (such as electric power systems^46,47^, photocatalysis^48^, bio-chemistry^49–51^, and gas leakage^52^) to drive significant advances in academic research and industrial development.

## Materials and methods

### Fabrication of TFBG sensor

A 18 mm-long TFBG was inscribed within a photosensitive glass single-mode optical fiber with a diameter of 125 μm (FIBERCORE PS1250/1500). By employing a KrF excimer laser emitted pulses with wavelength of 193 nm and passing through a phase mask with a period of 1098 nm, periodic interference fringes were generated and resulted in permanent refractive index modulation at fiber core. The resultant TFBG exhibited a core mode Bragg resonance at 1591 nm and tens of cladding mode resonances within the 1500–1600 nm spectral range. The tilt angle determines the wavelength separation between the higher order cladding modes and the Bragg mode. We selected a tilt angle of 16° to maximize the resonance amplitude of the interfacial modes in electrolyte with refractive indices of 1.37–1.39 (measured by a digital refractometer, Reichert 13940000). Finally, in order to obtain reflectance spectra, the optical fiber is cleaved a few millimeters downstream of the grating and coated with gold layer on the end face to increase broadband reflectivity. Another significant advantage of the reflective structure is that the cross-crosstalk in the evanescent field to strain on the TFBG is avoided, since no tension needs to be applied to keep the fiber straight.

### Sensing system

Figure 2b illustrates the optical fiber and electrochemical sensing system. It comprises a 1520–1620 nm broadband light source, a polarizer, a polarization controller, a circulator, a TFBG sensing probe and an optical spectrum analyzer. A battery test system is used to collect electrochemical data to correlate with the optical signals. Figure 2c shows a photograph of an entire optical fiber sensing probe, with a compact size of 18 mm in length and 125 μm in diameter. Figure 2d depicts the detailed configuration of the transparent quartz container for the lithium metal battery, which consists of two symmetrical Li metal electrodes in a liquid electrolyte and an optical fiber sensing probe is meticulously affixed one of the electrodes to sense the fast-changing of ionic concentrations at the electrolyte–electrode interface. Meanwhile, a horizontally arranged high-resolution microscope equipped with working distance objectives was used for continuous and real-time observation of the lithium dendrite growth and its morphology near the location of the fiber sensor. The cell was carefully sealed with paraffin wax at the top to keep it airtight while allowing optical and electrical connections to pass through.

### Principle and characteristics of the TFBG sensor

Fig. S1a displays the configuration of the TFBG. Input broadband light guided by the fiber core in the form of a single fundamental mode. The light interacts with a periodic array of tilted planes of raised refractive index inscribed inside the fiber core, generating a reflected core mode and reflected cladding modes. As the outer diameter of the fiber cladding is 125 μm, much larger than the light wavelength, numerous cladding modes can be excited, forming a fine comb of tens to hundreds of resonances in the transmission spectrum, as depicted in Fig. S1c. The tilt angle of the grating is a crucial parameter dictating the set of cladding modes that can be excited, spanning from tens to over one hundred nanometers. This tunability allows optimizing the sensor’s operation range to enhance performancce in the face of various ambient perturbations (Fig. S12).

### TFBG sensors in response to refractive index and temperature

As mentioned above, the TFBG can couple the incident core mode to generate a large number of reflected cladding modes, encompassing ghost modes, guided cladding modes, interface mode, and leaky cladding modes, sequentially appearing from long wavelengths to short wavelengths in the output spectrum (Fig. S1c). The guided cladding modes are tightly confined modes inside the fiber cladding because the effective refractive index (ERI) is larger than the SRI. In contrast, the ERIs of the leaky cladding modes are smaller than the SRI, resulting in no longer being guided by the cladding. The resonance dips of the leaky modes show only amplitude variations, without wavelength shifts, in response to SRI changes. The so-called “interface mode”, characterized by a sudden reduction in resonance amplitude, resides at the boundary between the guided and leaky modes. This interface mode features the maximum evanescent field penetration into the external medium, endowing it with the highest sensitivity to SRI. Hence, the interface mode of the TFBG is suitable for detecting external substance and composition changes near the outer surface of fiber cladding. Such changes in the vicinity of the outer surface of the cladding induce small variations in the SRI, and lead to both wavelength shift and amplitude variation of the interface mode. Thus, the SRI change can be tracked and the substance and composition changes can be interpreted.

The reflected core mode of a TFBG is tightly confined inside the fiber core, and identified by the resonance with the longest wavelength. This resonance exhibits the same temperature sensitivity (10 pm °C^−1^) as standard FBGs, inherently remaining insensitive to events occurring outside the cladding. Since all the core and cladding modes share identical temperature dependence and shift uniformly with temperature (refer to Fig. S13), compensating for the temperature cross-sensitivity in various sensing modalities becomes straightforward. This compensation is achieved by resolving relative wavelength offsets, such as those relative to the ghost mode, rather than focusing on absolute wavelengths. The ghost mode of TFBG represents a strongly guided cladding mode resonance, interacting prominently with the core–cladding interface but minor with the outside cladding boundary. This interaction arises due to the adjacency of their resonances to the core mode (about 2 nm away on the shorter wavelenght side). Similar to the core mode, the ghost modes exhibit comparable temperature sensitivity and are impervious to SRI changes. Consequently, the ghost mode can be effectively utilized for temperature measurements.

### Li anodes and electrolyte preparation

Four kind of Li anodes were used in this work. The bare, untreated Li anode was purchase from China Energy Lithium Co. Ltd. (Tianjin, China). The Li–Al anode comprised of a Li metal layer and a Li_*x*_Al alloy layer, which was obtained by machine-pressing the stacked bare Li anode and 1 μm-thick aluminum foil under a pressure of 10 MPa^53^. The Li–Li_3_PO_4_ anode consisted of a Li metal layer and a 1 μm-thick Li_3_PO_4_ layer, which prepared by soaking bare Li metal in DMSO with 0.2 wt% H_3_PO_4_ solution for 2 min^9^. The Li–Cu anode consisted of a Li metal layer and a 1 μm-thick layer of Cu nanoparticles, which was fabricated by spin coating a DMSO solution with 1 wt% copper nanoparticles onto the bare Li anode^45^. The anodes used for the symmetrical cells with TFBG sensors were fabricated by pressing ~100 μm Li metal layer onto a 300 μm-thick copper sheet substrate, followed by the treatment procedures as described above. To prepare the electrolytes, 4 mol L^−1^ lithium hexafluorophosphate in EC, EMC, DMC (1:1:1, v/v/v, respectively) was prepared (denoted in the text as 4 M LiPF_6_ EC:EMC:DMC).

### Cell assembly and electrochemical test

The experimental symmetrical cells with TFBG were fabricated in sealed quartz electrolytic cells, and 8 mL 4 M LiPF_6_ EC:EMC:DMC electrolyte was injected to submerge the electrode. The coin type symmetrical cells were assembled with 4 M LiPF_6_ EC:EMC:DMC electrolyte in an Ar-filled glove box, and the Li anode was 450 μm thick with a diameter of 15.6 mm. Five drops (approximately 150 μL) of electrolyte were added to each cell. Galvanostatic charge–discharge tests were performed using a LAND CT2001A cell test system (Land Co. Ltd., China). The symmetrical cells in the quartz electrolytic cell and in the 2032-coin cells were cycled at 1 mA cm^−2^ for 0.5 h, and 0.5 mA cm^−2^ for 1 h, respectively.

### Characterization

SEM measurements were carried out using a Hitachi S-4800 field emission scanning electron microscope equipped with EDS. XPS measurements were conducted using a Phi X-tool XPS instrument. The anode morphology was examined under an optical microscope (Aurora-II, Thermo Fisher Scientific). In situ observation of the lithium dendrite growth process was examined under an optical microscope (NAVITAR AMETEK).

### Supplementary information

Supporting document

Video for Li dendrite formation

## Data Availability

The data that support the plots within this paper and another finding of this study are available from the corresponding author upon reasonable request.
