# Supplementary material for: Operando monitoring of dendrite formation in lithium metal batteries via ultrasensitive tilted fiber Bragg grating sensors
Source: Light Sci Appl. 2024 Jan 22;13:24. doi: 10.1038/s41377-023-01346-5 (PMC10803745; doi:10.1038/s41377-023-01346-5)
Supplement: Supplementary file 1 — Supporting document [file 41377_2023_1346_MOESM1_ESM.docx]

**Supplementary Information**

***Operando* monitoring of dendrite formation in lithium metal batteries via ultrasensitive tilted fiber Bragg grating sensers**

Xile Han^1†^, Hai Zhong^2†^, Kaiwei Li^1, 3†^, Xiaobin Xue^1†^, Wen Wu^1^, Nan Hu^1^, Xihong Lu^4^, Jiaqiang Huang^5*^, Gaozhi Xiao^6*^, Yaohua Mai^2*^, Tuan Guo^1*^

^1^ *Institute of Photonics Technology, Jinan University, Guangzhou 510632, China*

*^2^ Institute of New Energy Technology, College of Information Science and Technology, Jinan University, Guangzhou 510632, China*

*^3^ Key Laboratory of Bionic Engineering (Ministry of Education), Jilin University, Changchun 130022, China*

*^4^ The Key Lab of Low-Carbon Chemistry & Energy Conservation of Guangdong Province, School of Chemistry, Sun Yat-Sen University, Guangzhou 510275, China*

*^5^ Sustainable Energy and Environment Thrust, The Hong Kong University of Science and Technology (Guangzhou), Nansha, Guangzhou, Guangdong, 511400, China*

*^6^ Advanced Electronics and Photonics Research Centre, National Research Council of Canada, Ottawa K1A 0R6, Canada*

^*^Correspondence and requests for materials should be addressed to Jiaqiang Huang (seejhuang@ust.hk) or Gaozhi Xiao (george.xiao@nrc-cnrc.gc.ca) or Yaohua Mai (yaohuamai@jnu.edu.cn) or Tuan Guo (tuanguo@jnu.edu.cn)

^†^These authors contributed equally: Xile Han, Hai Zhong, Kaiwei Li, Xiaobin Xue.

**
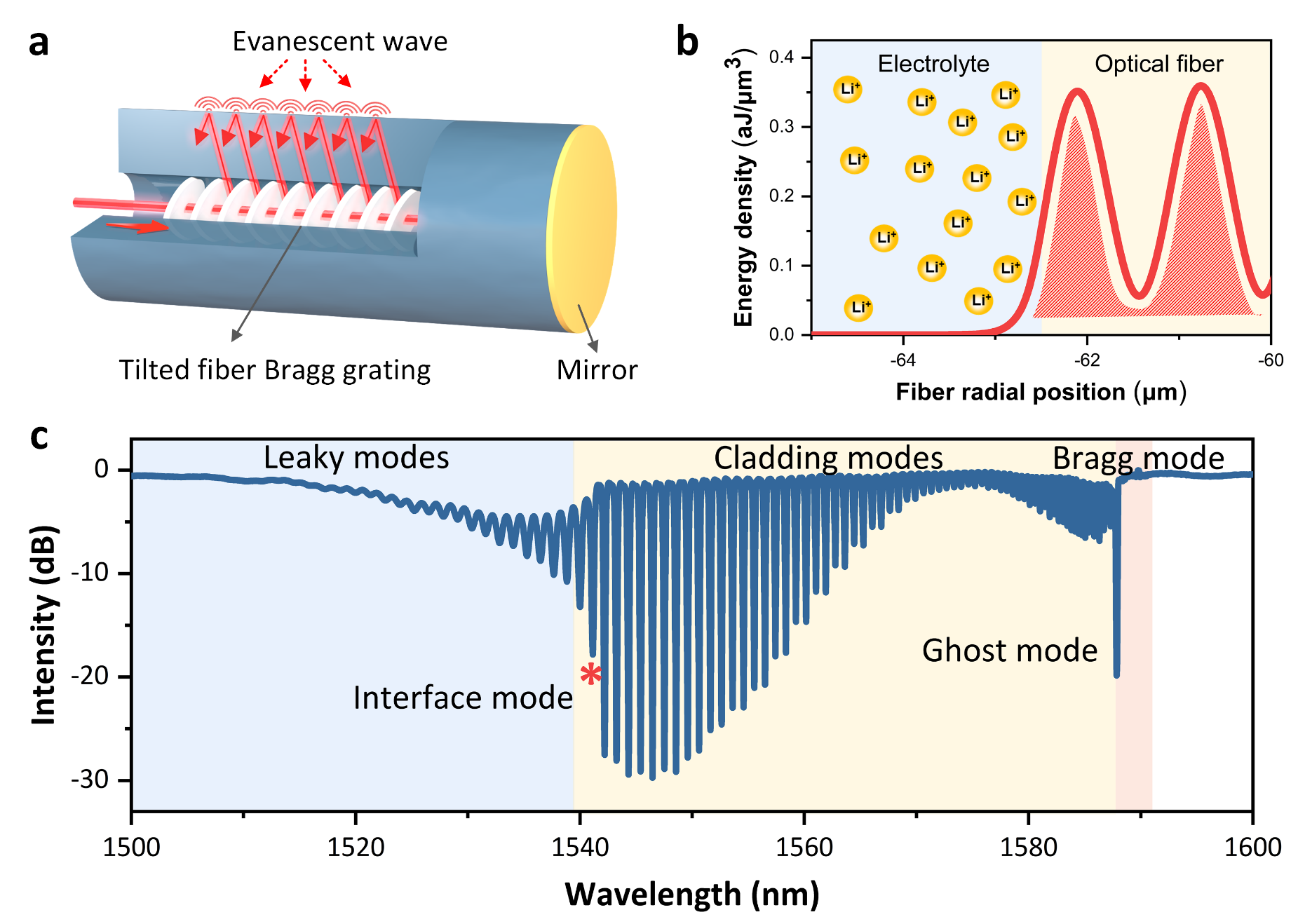
**

**Fig. S1** **a** Schematic of the configuration of a reflective optical fiber sensor for *in situ* monitoring of ionic concentrations. The tilted fiber Bragg grating (TFBG) is formed by a periodic array of tilted planes of increased refractive index inscribed in the fiber core. Light injected into the fiber core is resonantly coupled into the cladding and its evanescent field extends into the surrounding electrolyte. A mirror on the fiber tip surface reflects optical signals back to the upstream fiber for interrogation. **b** Simulated electric mode field profiles of high efficiency evanescent wave over fiber surface excited by polarized light in the fiber core. **c** The typical comb reflection spectrum of a 16° TFBG in medium with a refractive index of 1.37.


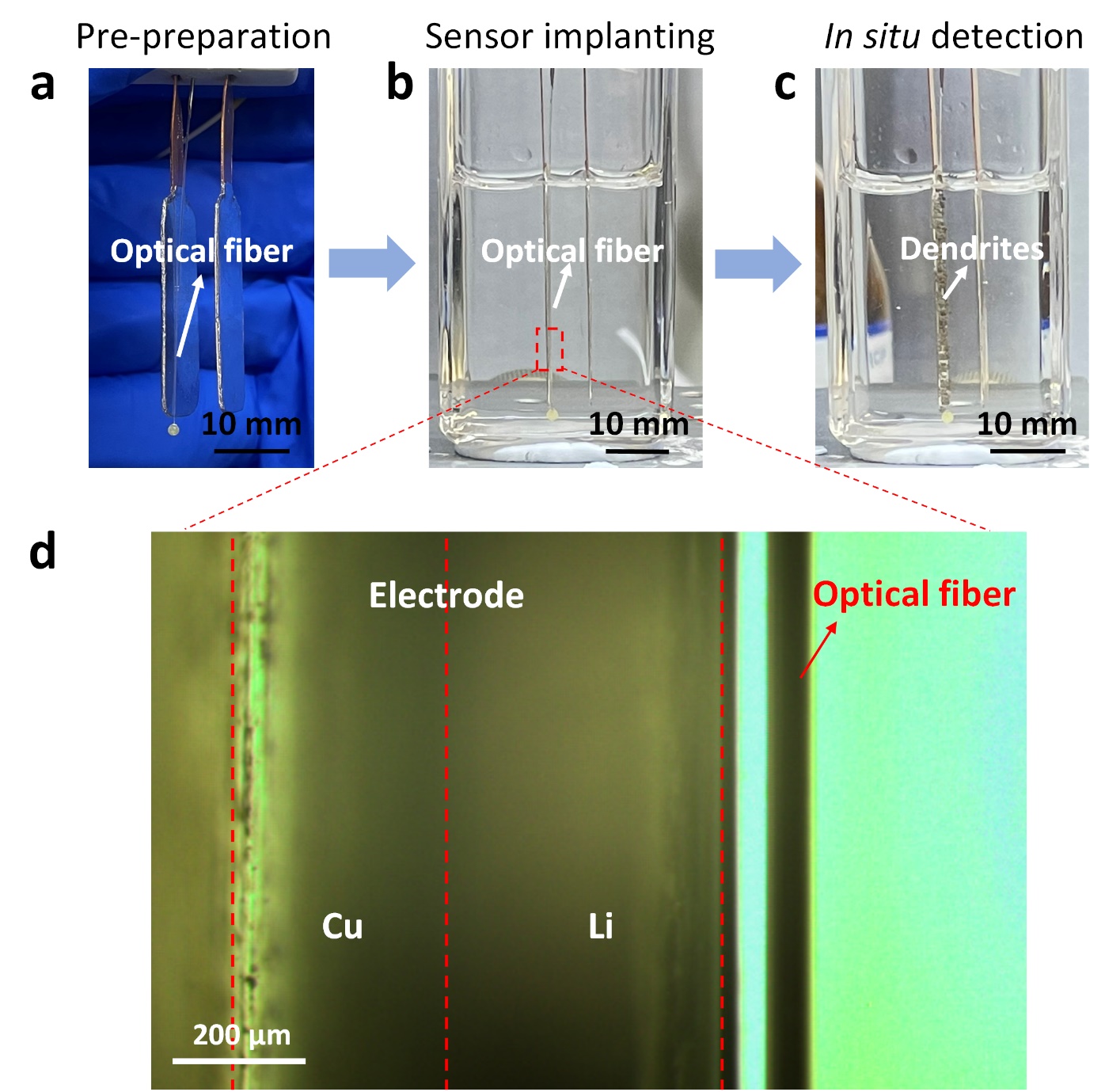


**Fig. S2 Dark field optical microscope** **images of lithium battery and cross-section of the fiber and electrodes. a-c** The optical fiber *in situ* detection system for lithium metal battery. **d** Local zoom view of lithium metal anode: the optical fiber sensor is attached to the surface of the electrode.


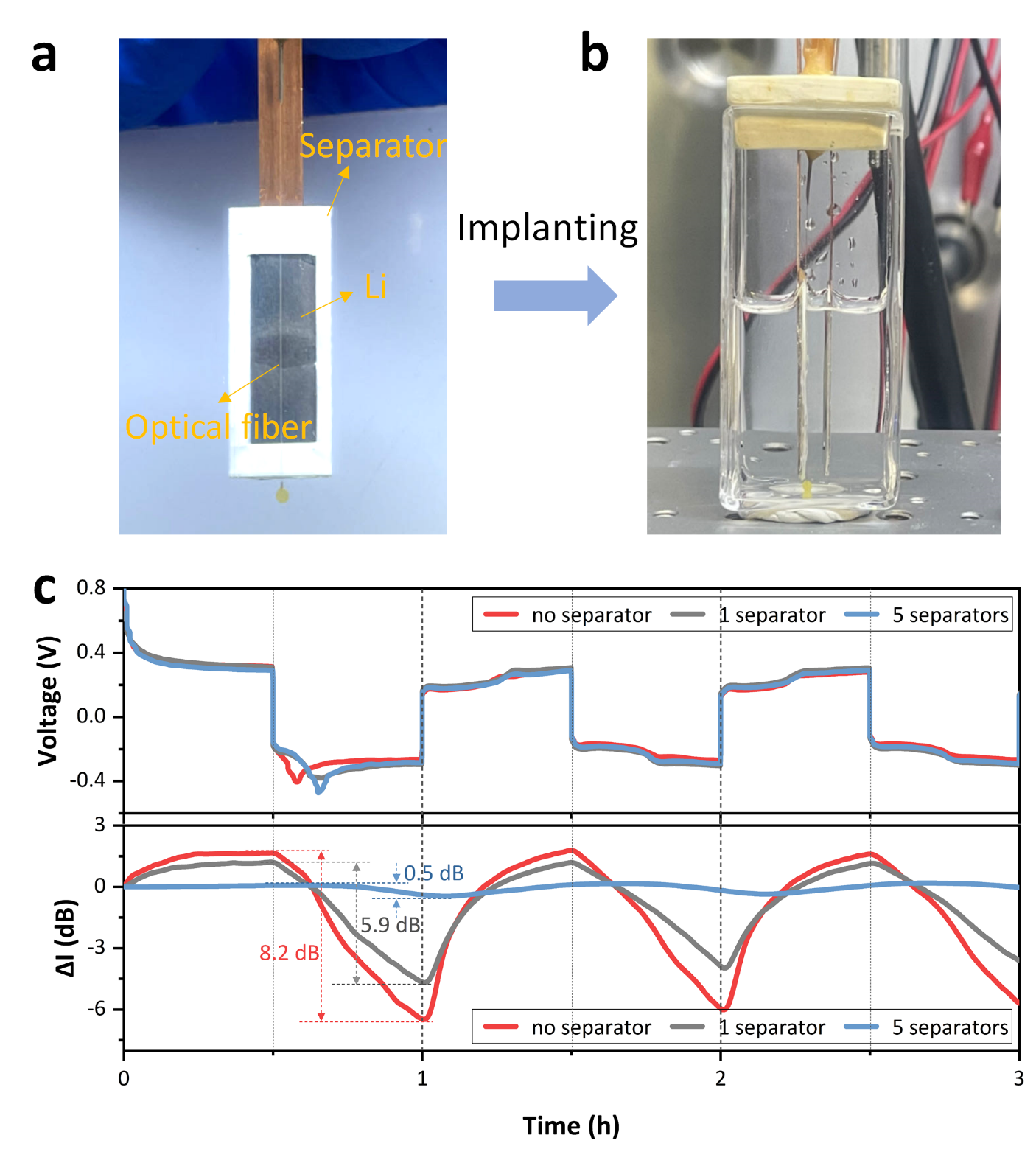


**Fig. S3 Spatial-temporal evolution characteristics of species concentration. a** The separators are placed between the optical fiber and the lithium metal electrode. **b** *in situ* detection system for spatial-temporal evolution of species concentration. **c** periodic voltage variation with time (top), the corresponding periodic variation of interface mode amplitude of the implanted TFBG (bottom) in charge-discharge cycling tests of the lithium battery with and without separators.

As shown in **Fig. S3a**, we utilize the separator (Celgard, 2500) to precisely control the distance between the optical fiber sensor and the lithium metal electrode to detect spatial distribution of species concentration. The distance is controlled to 25 μm and 125 μm by 1-layer and 5-layer separators, respectively. The optical signal response intensity of the optical fiber sensor is modulated by a change of refractive index of the electrolyte caused by the species concentration. Therefore, as a distance between the optical fiber sensor and the lithium electrode increases to 125 μm, the change amplitude of the optical signal response intensity decreases from 8.2 dB to 0.5 dB (**Fig. S3b**), indicating a more intense change in species concentration at the electrode surface.


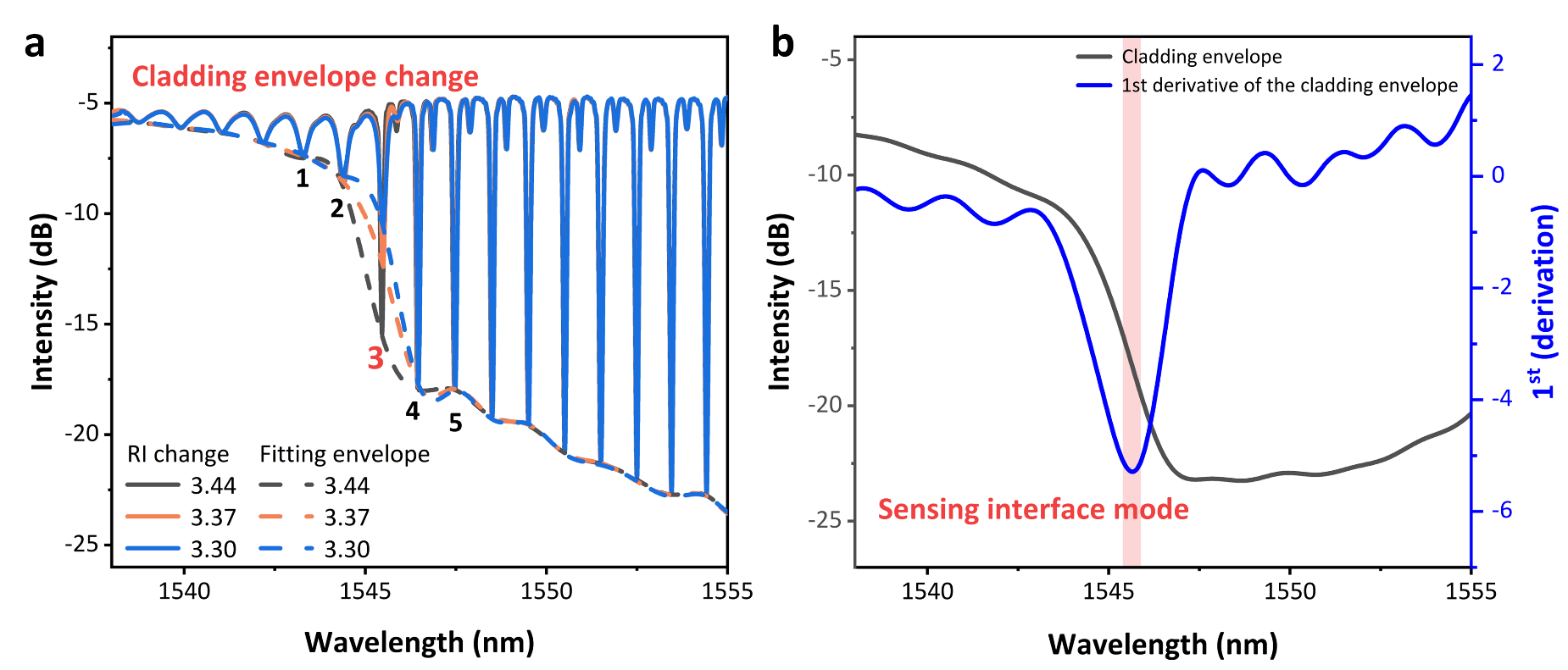


**Fig. S4 The principle of selection of the most sensitive interface mode. a** The measurement of a 16° TFBG as a function of external refractive index (solid line). The corresponding fitting envelope of the attenuated cladding mode resonances is moving towards lower intensity (dash line). **b** the fitted envelope of the cladding (black line) and its 1st derivative (blue line).

The resonance with the wavelength of 1545.7 nm is the most attenuated cladding modes, which is most sensitive to external refractive index perturbation (hybrid plasmon guided mode) and is widely used for sensing [1]. The corresponding analysis is shown in **Fig. S4a**, in which all attenuated envelope modes covering the attenuation region are labeled from 1 to 5, and the corresponding fitted envelope decays towards lower intensity when external refractive index reduces. If we perform a first derivative for the fitted envelope (**Fig. S4b**), the cladding mode marked by number 3 shows the largest slope and therefore the most sensitive to RI changes, especially for very slight surface RI perturbations during the charging/ discharging process inside batteries.


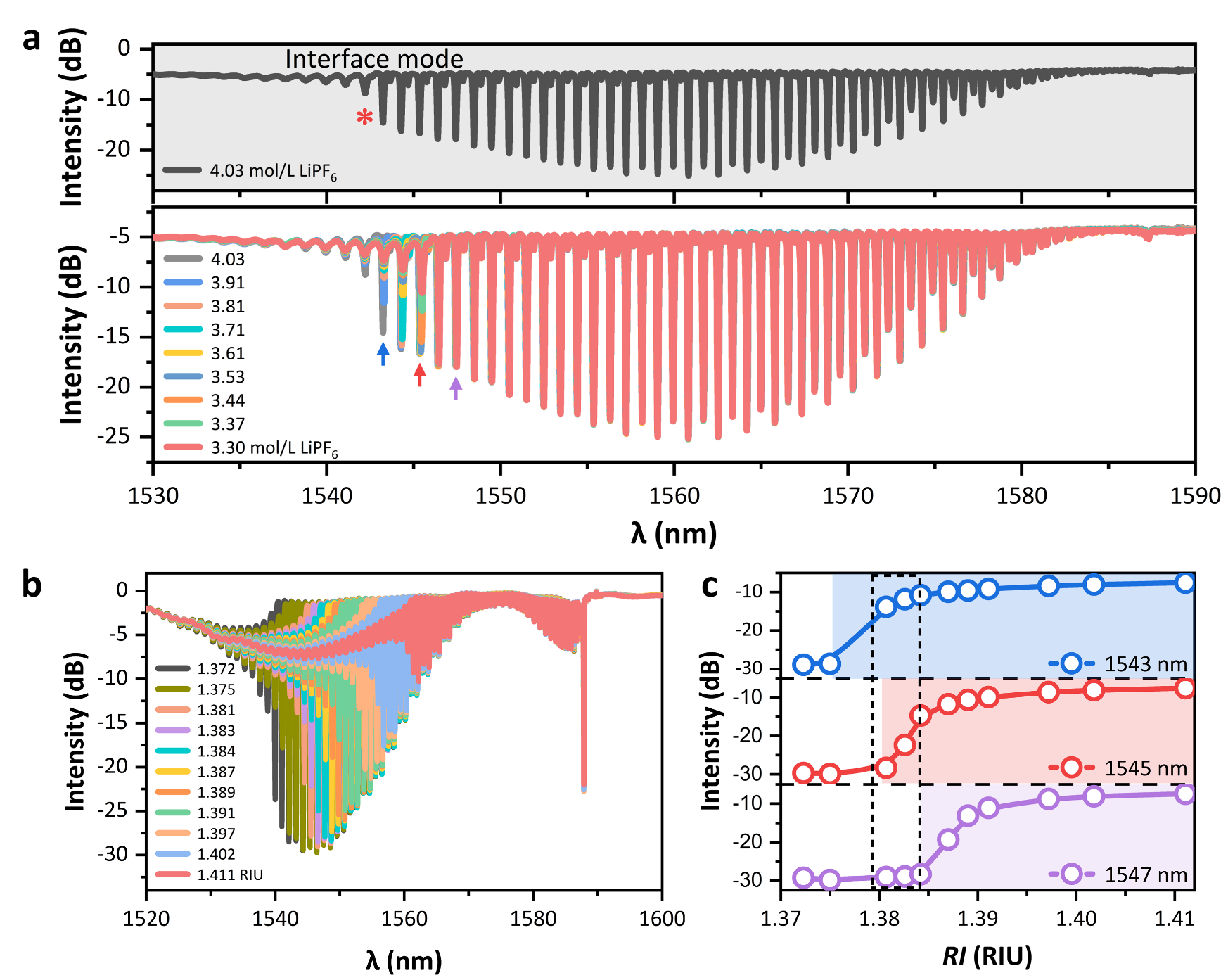


**Fig. S5 Refractive index response of TFBG spectra.** **a** The spectral response of a TFBG with a tilt angle of 16° in LiPF_6_ EC:EMC:DMC with concentrations ranging from 3.3 to 4.03 M. The top graph shows the individual spectrum in the 4.03 M LiPF_6_ electrolyte, where the interface mode is marked by a red "*"; The wavelength of the interface mode varies with the electrolyte. As the electrolyte concentration decreases, the interface mode shifts toward longer wavelengths, and its amplitude decreases. The blue, red, and purple arrows indicate three candidate interface modes. The intensities of these three modes are shown in **c**. **b** The spectral responses of a TFBG with a tilt angle of 16° in glycerol solution with a refractive index range of 1.372-1.411. The wavelength of the interface mode of TFBG spectrum increases with increasing refractive index. The ghost mode in **Fig. S5b** is different from **Fig. S5a** due to the TFBG fabrication. **c** Amplitude of the interface modes at the wavelengths of 1543 nm, 1545 nm, and 1547 nm as a function of the refractive index. The dashed rectangle identifies the useful measurement range of the studied example around 1.38 RIU.


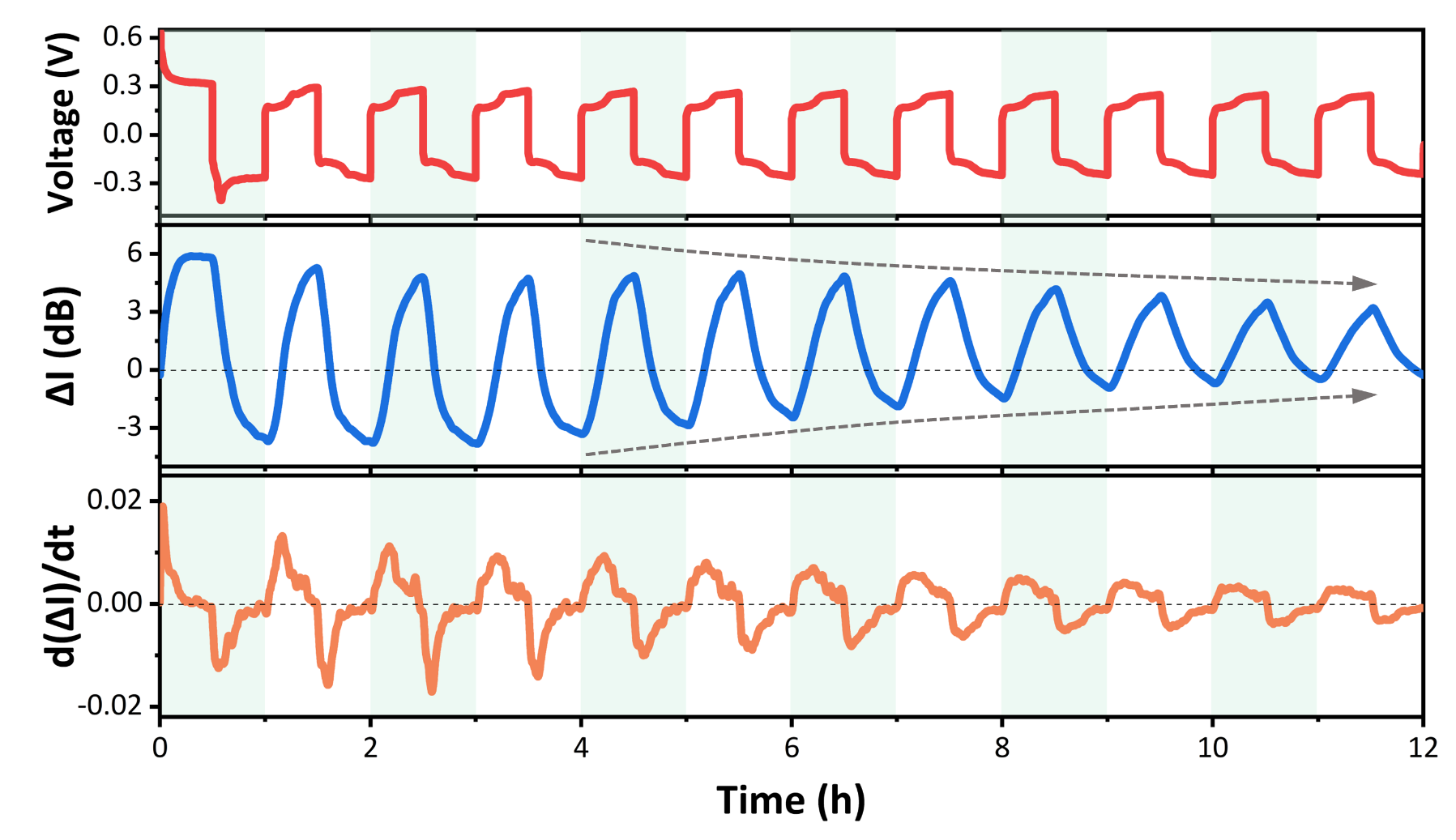


**Fig. S6** The voltage curve (red), interface mode intensity curve (blue) and differential of light intensity (orange) of Li/Li symmetric cells at a current density of 1 mA cm^-2^ during repeated charge-discharge cycling.


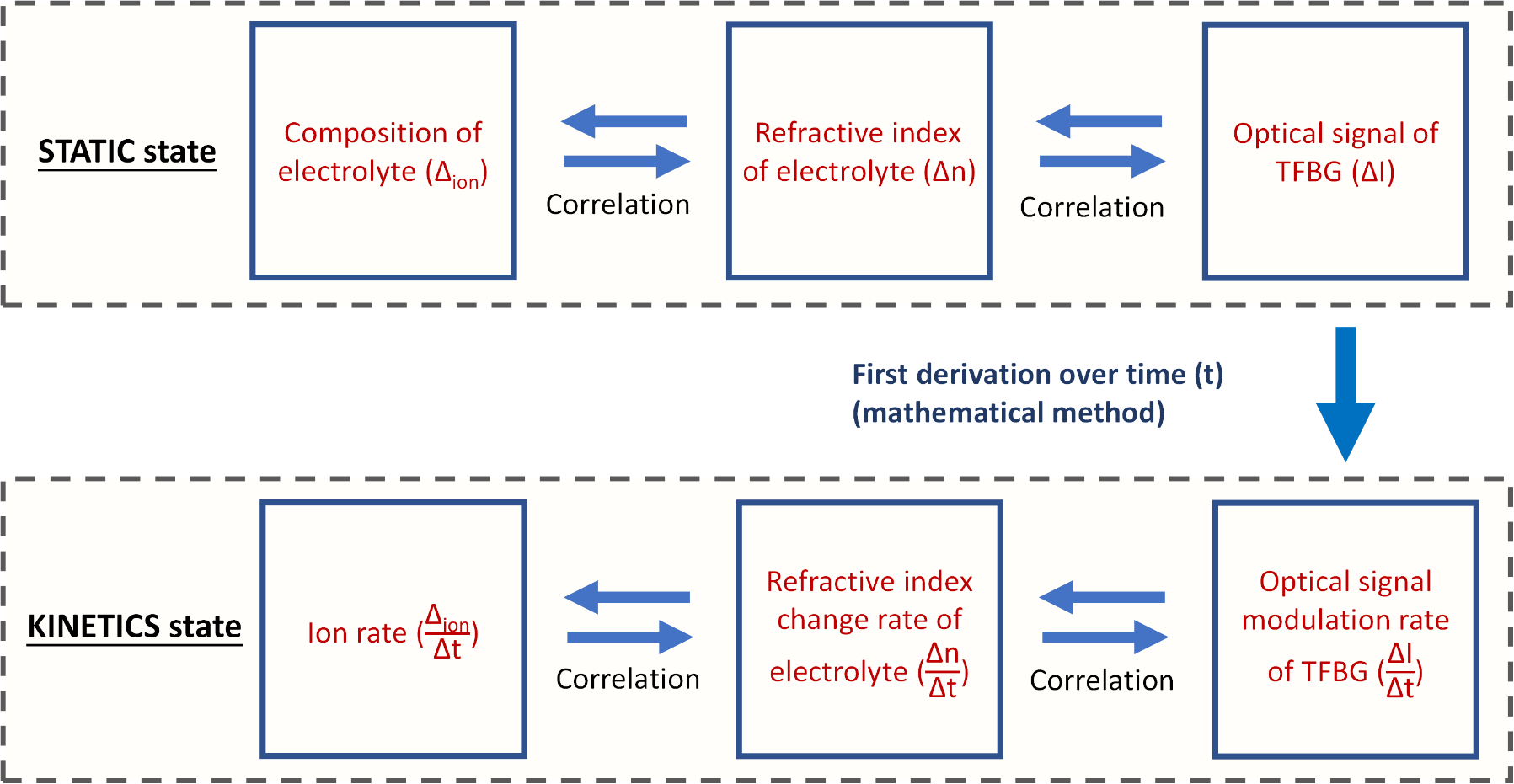


**Fig. S7.** Schematic diagram of STATIC and KINECTIC state for the relation between optical sensor and electrolyte.

The correlation between ion rate and the time derivative of the optical signal is best appreciated in Fig. S7. Firstly, the optical signal response of TFBG (∆I) corresponds to changes in the refractive index of the electrolyte (∆n), induced by variations in ion concentration (∆_ion_). Secondly, the amplitude changes of the interface mode of TFBG can be used to characterize the changes in the ion concentration at the electrode-electrolyte interface. Thus, the temporal derivative of the optical signal (∆I/∆t) unveils the rate of change in ion concentration(∆_ion_/∆t). Benefiting from the fast response time (0.1 ms/ nm) and super fine spatial resolution (sub-μm-scale) of the proposed TFBG optical fiber sensor, we reveal the correlation between static ion concentration & ion transport kinetics at electrolyte-electrode interface and the optical signal of the fiber sensor, which can be used to characterize the mass transport characteristics and chemical stability of different SEI layers.

**
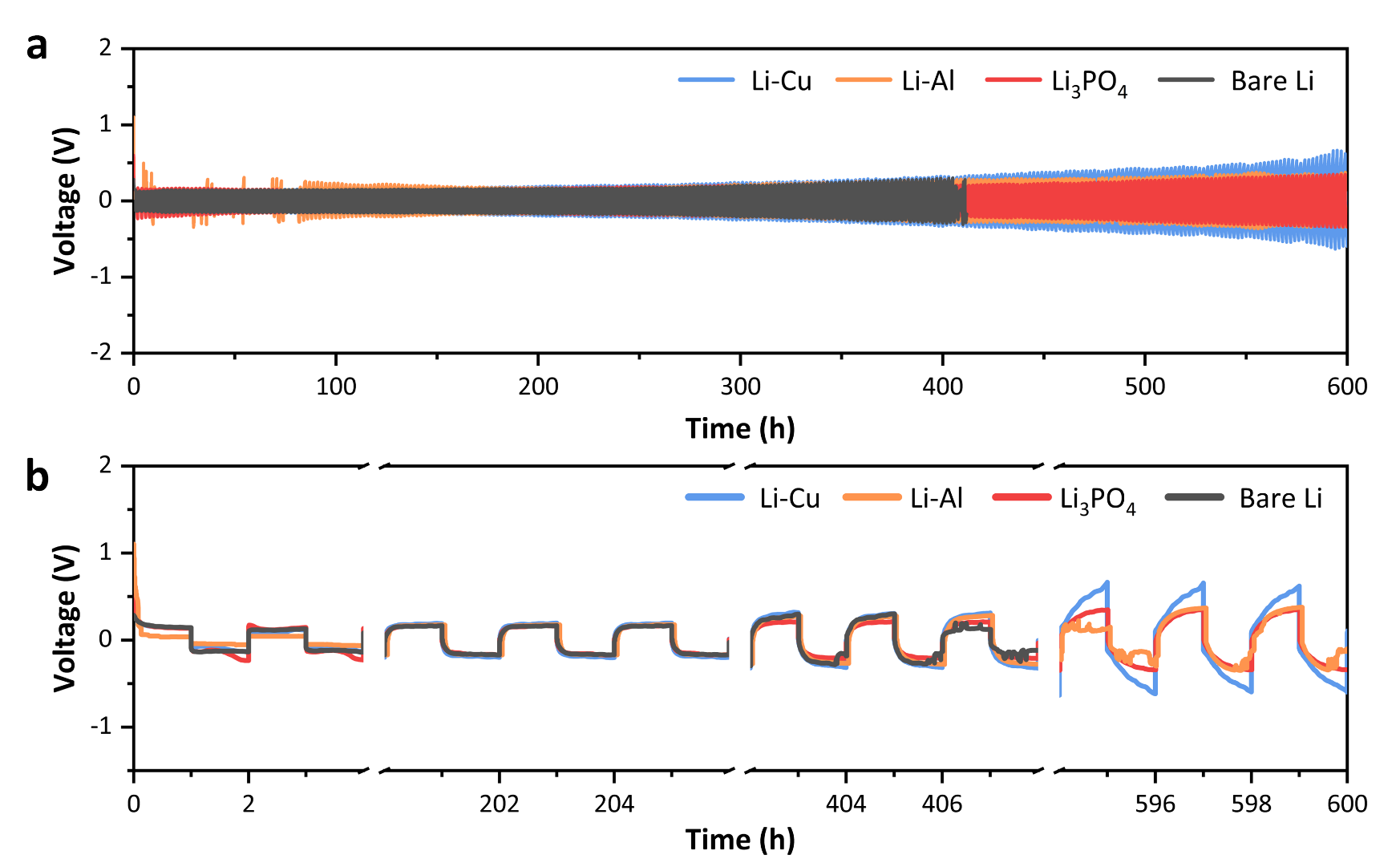
**

**Fig. S8 a** Cycling comparison for Li-Cu/Li-Cu, Li-Al/Li-Al, Li-Li_3_PO_4_/Li-Li_3_PO_4_, and Li/Li symmetric cells at a current density of 0.5 mA cm^-2^ for the area capacity of 0.5 mAh cm^-2^; **b** Horizontally expanded views of the voltage profiles over selected time intervals.

The effects of surface engineered Li metal anodes were evaluated in symmetrical cell coin cells, and the galvanostatic cycling performance of these cells was studied at a current density of 0.5 mA cm^-2^ with an area capacity of 0.5 mAh cm^-2^. **Fig. S8a** demonstrates that the Li-Li_3_PO_4_/Li-Li_3_PO_4_ symmetrical cell exhibits stable cycling with slight increase in voltage polarization over time. By contrast, the Li-Cu/Li-Cu symmetrical cell exhibited gradual voltage polarization augmentation, and Li/Li and Li-Al/Li-Al symmetrical cells showed significant reductions in voltage polarization after approximately 408 and 594 h of cycling. The detailed voltage profiles of the cells at selected cycle numbers are displayed in **Fig. S8b**. Differences can be observed over the ranges of 404-408 and 592-596 h, indicating that the Li-Li_3_PO_4_/Li-Li_3_PO_4_ symmetrical cell exhibited a normal Li plating/stripping voltage; the Li-Cu/Li-Cu symmetrical cell showed a remarkable increase in voltage polarization; and Li/Li and Li-Al/Li-Al symmetrical cells likely short circuited.


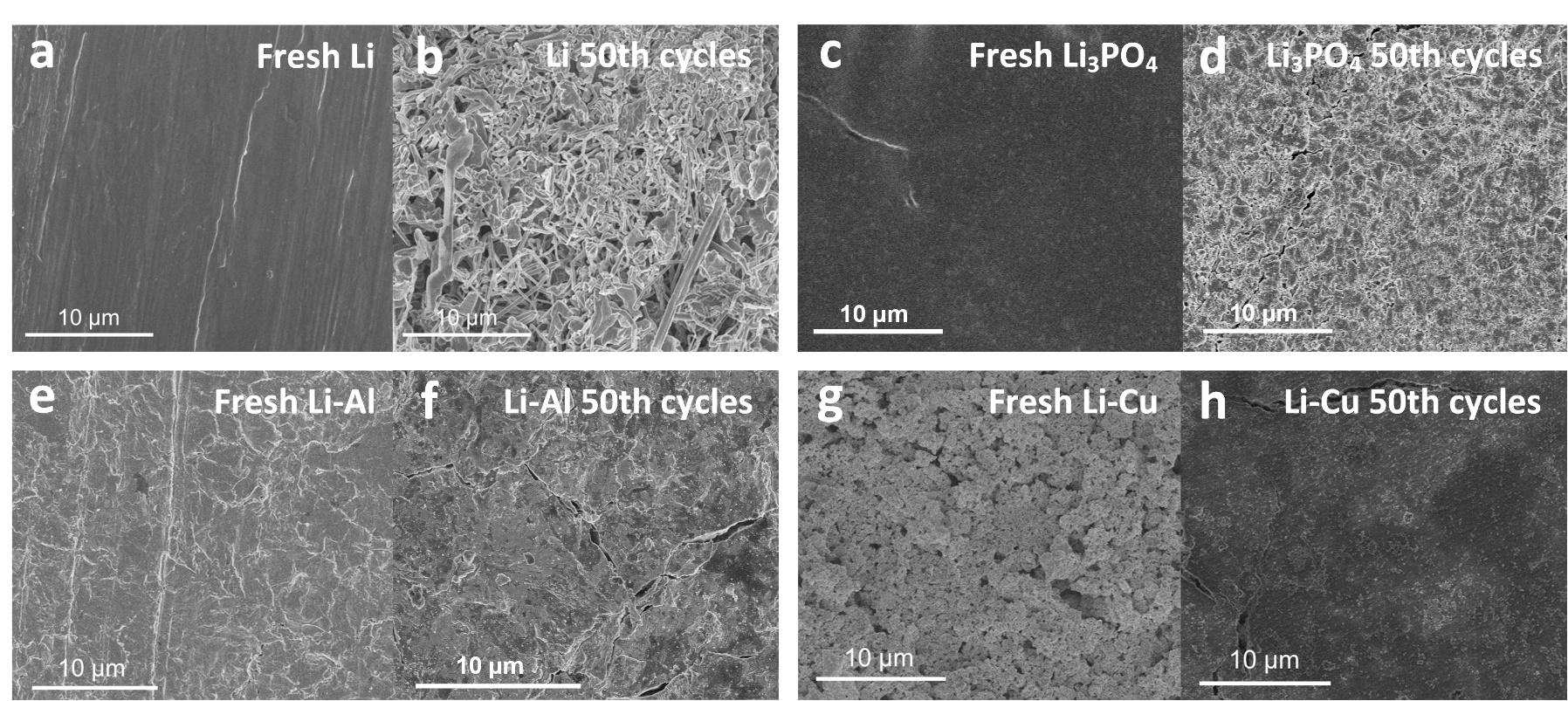


**Fig. S9** Typical SEM images at the surfaces of **a** fresh Li anode, **c** fresh Li-Li_3_PO_4_ anode, **e** fresh Li-Al anode, **g** fresh Li-Cu anode; **b, d, f, h** corresponding anodes after the 50 cycles.

The corresponding morphologies of four anodes after 50 cycles are shown in **Fig. S9**, alongside the fresh anodes used for comparison. The initially smooth bare Li anode displayed a typical lithium dendrite growth morphology after 50 cycles. However, the Li-Li_3_PO_4_ anode and Li-Al anode maintained their smooth surfaces and no dendrite formation could be observed. It is suggested that Li_3_PO_4_ layer and Li_x_Al layers were able to greatly suppress the dendrite growth. Interestingly, the porous surface of the Li-Cu anode became smoother after 50 cycles, which we explain by the decomposed electrolyte compounds filling in the pores. The results confirmed that a fabricated artificial SEI layer on the Li anode is able to suppress lithium dendrite formation.


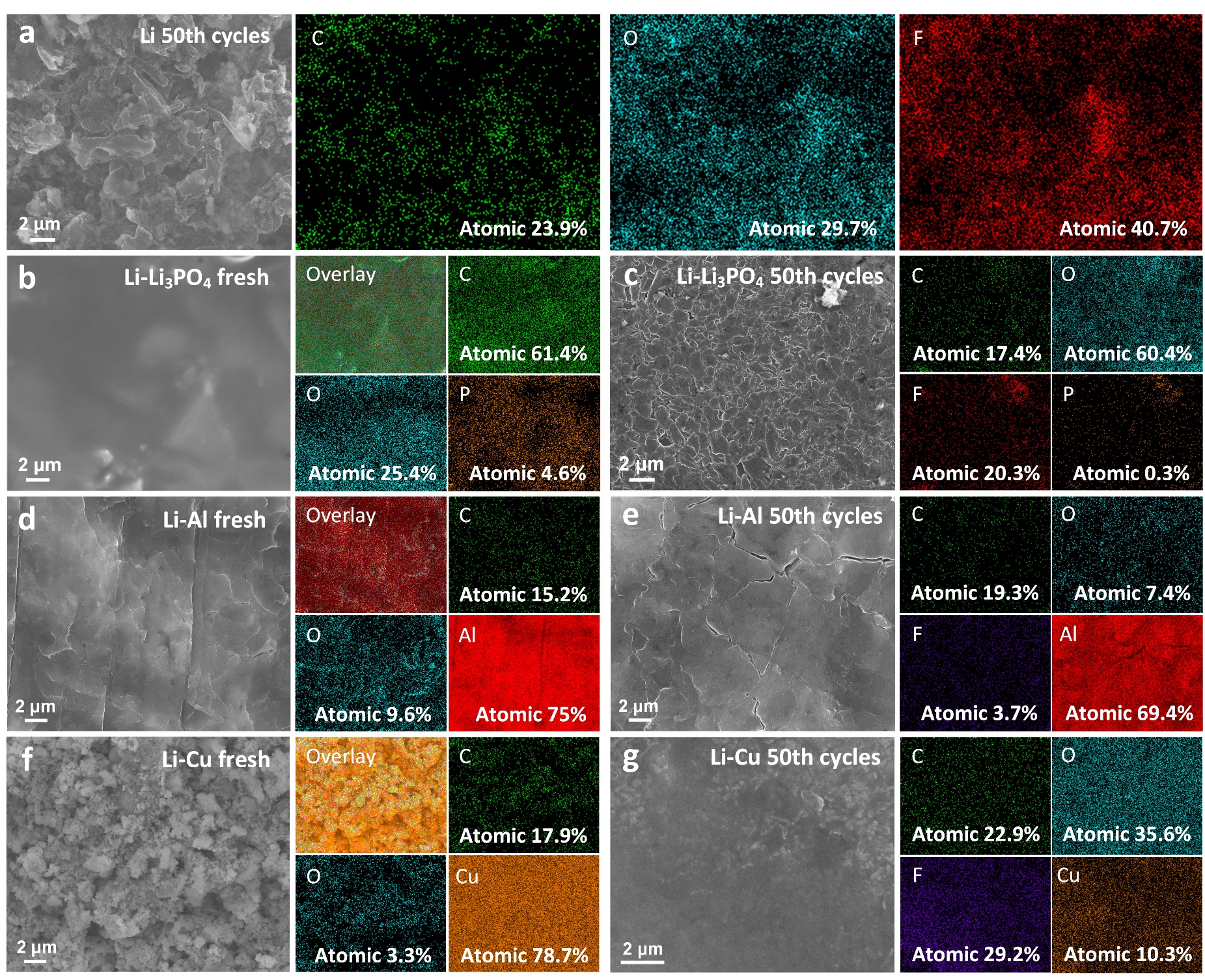


**Fig. S10 SEM images and element maps of four Li anodes before and after 50 cycles**. SEM images of **a** Li anode, **c** Li-Li_3_PO_4_ anode, **e** Li-Al anode, **g** Li-Cu anode after 50 cycles, and their corresponding energy dispersive spectroscopy (EDS) maps. SEM images of **b** fresh Li-Li_3_PO_4_ anode, **d** fresh Li-Al anode, **f** fresh Li-Cu anode, and their corresponding EDS maps.

The SEM images and their corresponding element maps are presented in **Fig. S10**. The bare Li anode images showed only noise due to impurity content and therefore have not been displayed. The typical elements, P, Al and Cu, were uniformly distributed on the surface of fresh Li-Li_3_PO_4_, Li-Al and Li-Cu anodes, respectively. The results suggest that the three of artificial SEI layers uniformly coated the Li metal layer. The uniformed distribution fluoride can be observed after Li anode cycles. In addition, the uniformed distribution of typical elemental of P, Al and Cu also can be observed on their surface of anode after cycles.


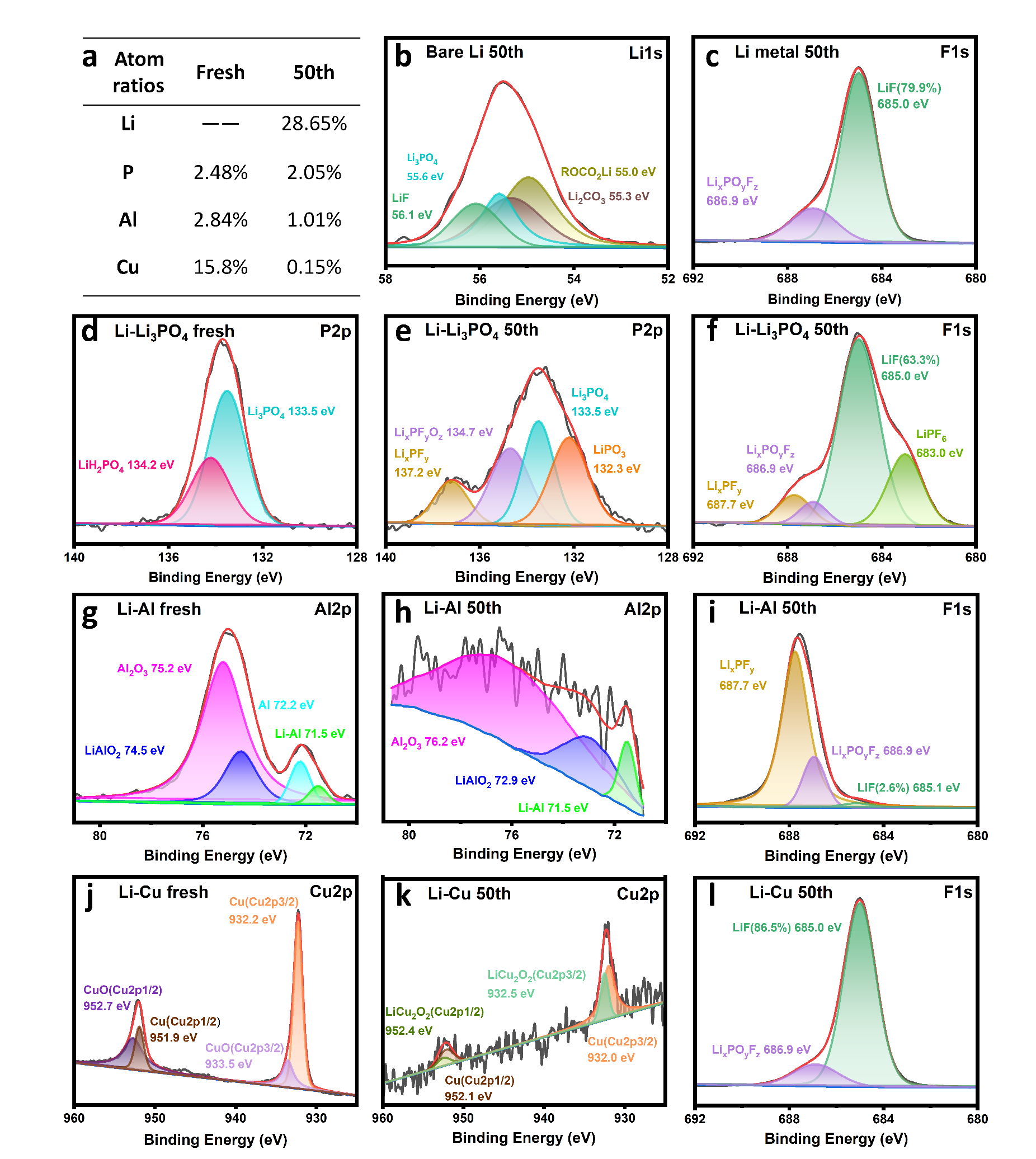


**Fig. S11 X-ray photoelectron spectroscopy (XPS) spectra of four Li anodes before and after 50 cycles**. **a** Typical elemental ratios (atom%) of four Li anodes before and after cycles; **b** Li 1s and **c** F 1s region of bare Li anode after 50 cycles; **d** P 2p region of Li-Li_3_PO_4_ anode; **e** P 2p and **f** F 1s region of Li-Li_3_PO_4_ anode after 50 cycles; **g** Al 2p region of Li-Al anode; **h** Al 2p and **i** F 1s region of Li-Al anode after 50 cycles; **j** Cu 2p region of Li-Cu anode; **k** Cu 2p and **l** F 1s region of Li-Cu anode after 50 cycles.

The ex-situ XPS of lithium anodes before and after cycles were collected to attest to the changes of chemical state and composition of the abovementioned SEI. The bare Li anode was not tested due to the impurity content of bare Li. **Fig. S11a**, shows the typical Li elemental ratios of bare Li anode and Cu elemental ratios of Li-Cu anode are given an obvious change before and after cycles, which result in electrolyte deposition of decomposed compounds on the anode surface. By the contrast, the typical P elemental ratios of the Li-Li_3_PO_4_ anode and Al elemental ratios of the Li-Al anode decreased only slightly, which suggests that the Li metal plating reaction occurs underneath the artificial SEI layer. XPS spectra of the corresponding anodes (**Fig. S11b-l**) serve to further demonstrate species on the anode surface of anode. All of the anodes showed fluoride deposition after 50 cycles, but there are some differences in chemical composition. The main fluoride species are LiF and Li_x_PO_y_F_z_ on the bare Li anode after 50 cycles (**Fig. S11c**), and the similar fluoride species formed on the Li-Cu anode (**Fig. S11l**), while the fluoride-containing compounds on the Li-Li_3_PO_4_ anode are LiF, LiPF_6_, Li_x_PO_y_F_z_, and LiP_x_F_y_ (**Fig. S11f**). On the Li-Al anode the fluoride compounds are LiP_x_F_y_, Li_x_PO_y_F_z_, and LiF (**Fig. S11i**). Subsequently, the chemical state of typical elemental species was also investigated by deconvolution of their corresponding elemental peaks. From **Fig. S11d** and **e**, the Li_3_PO_4_ and LiH_2_PO_4_ compounds on the fresh Li-Li_3_PO_4_ anode were identified as Li_3_PO_4_, LiPO_3_, Li_x_PO_y_F_z_, and LiP_x_F_y_. The LiH_2_PO_4_ has disappeared due to it having completely reacted with lithium metal after 50 cycles. The Al_2_O_3_, LiAlO_2_, Al, Li_x_Al compounds on the fresh Li-Al anode (**Fig. S11g**) changed to Al_2_O_3_, LiAlO_2_, Li_x_Al compounds (**Fig. S11h**). The incompletely lithiated Al at the Li-Al anode fabricated could be continuous reacted with lithium metal until completely lithiation. The Cu and CuO compounds on the fresh Li-Cu anode (**Fig. S11j**) changed to Cu and LiCu_2_O_2_ compounds (**Fig. S11k**).


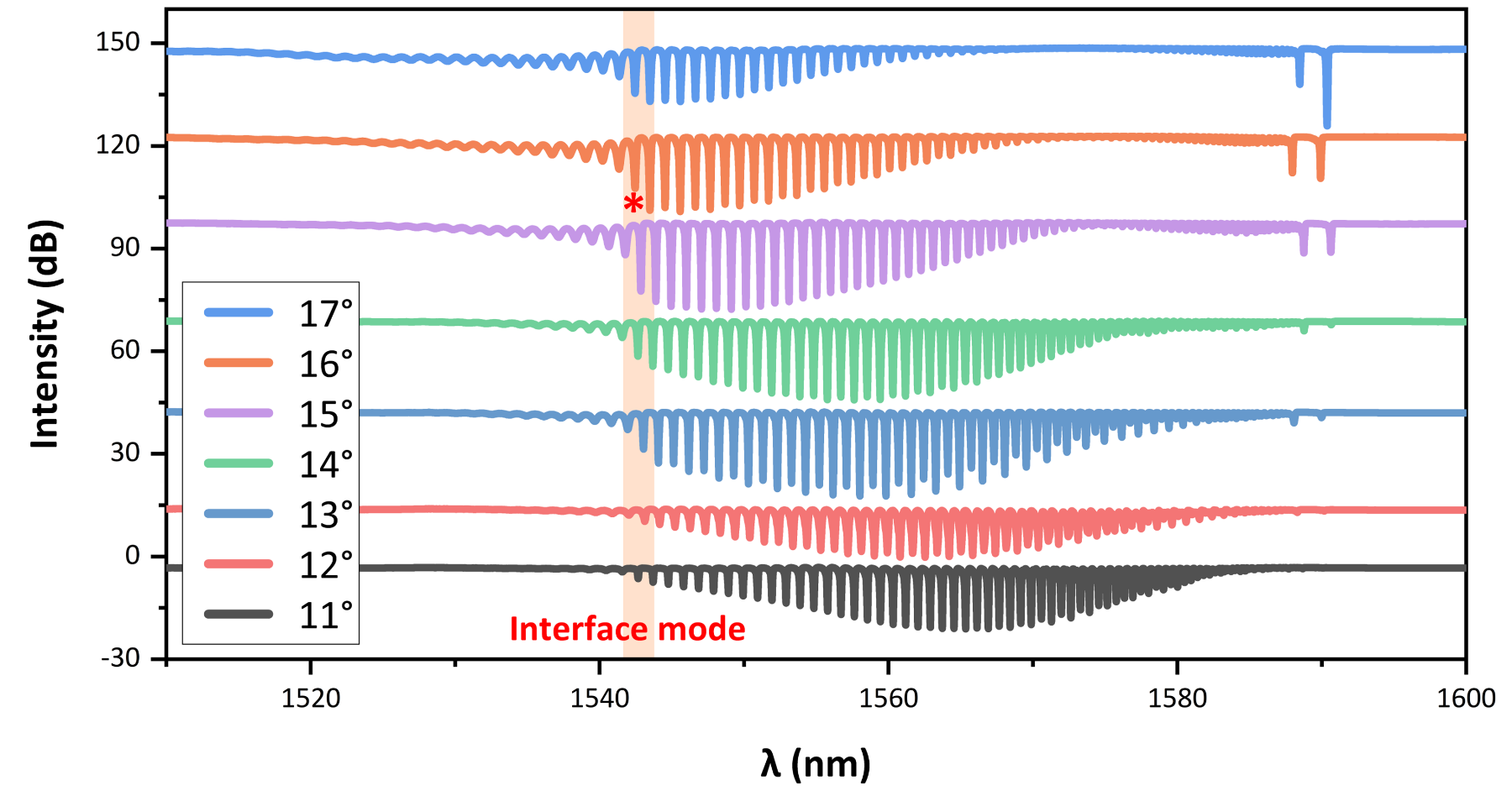


**Fig. S12** Simulated spectra for TFBGs immersed in electrolyte of refractive index 1.378 for tilt angles 11° to 17°.

The selection of the optimum tilting angle of the fiber grating depends on the refractive index of the electrolyte. The maximum sensitivity is obtained when the cladding modes have effective indices close to the index of the electrolyte. In Li metal batteries, we measured the electrolyte refractive index to be 1.378, which varies by less than ±0.05 during charging/discharging cycles. For a grating with a Bragg wavelength near 1589 nm, cladding modes with effective indices near 1.378 occur in the spectral region between 1540 and 1550 nm. In order to maximize the amplitudes of the cladding modes at these wavelengths, the tilt angle was selected based on simulations of TFBG spectra, as shown in **Fig. S12**. They confirm that the maximum of the envelope of resonance amplitudes moves towards shorter wavelengths as the tilt angle increases and that the interface mode location falls within the desired spectral window for a tilt angle around 16°.


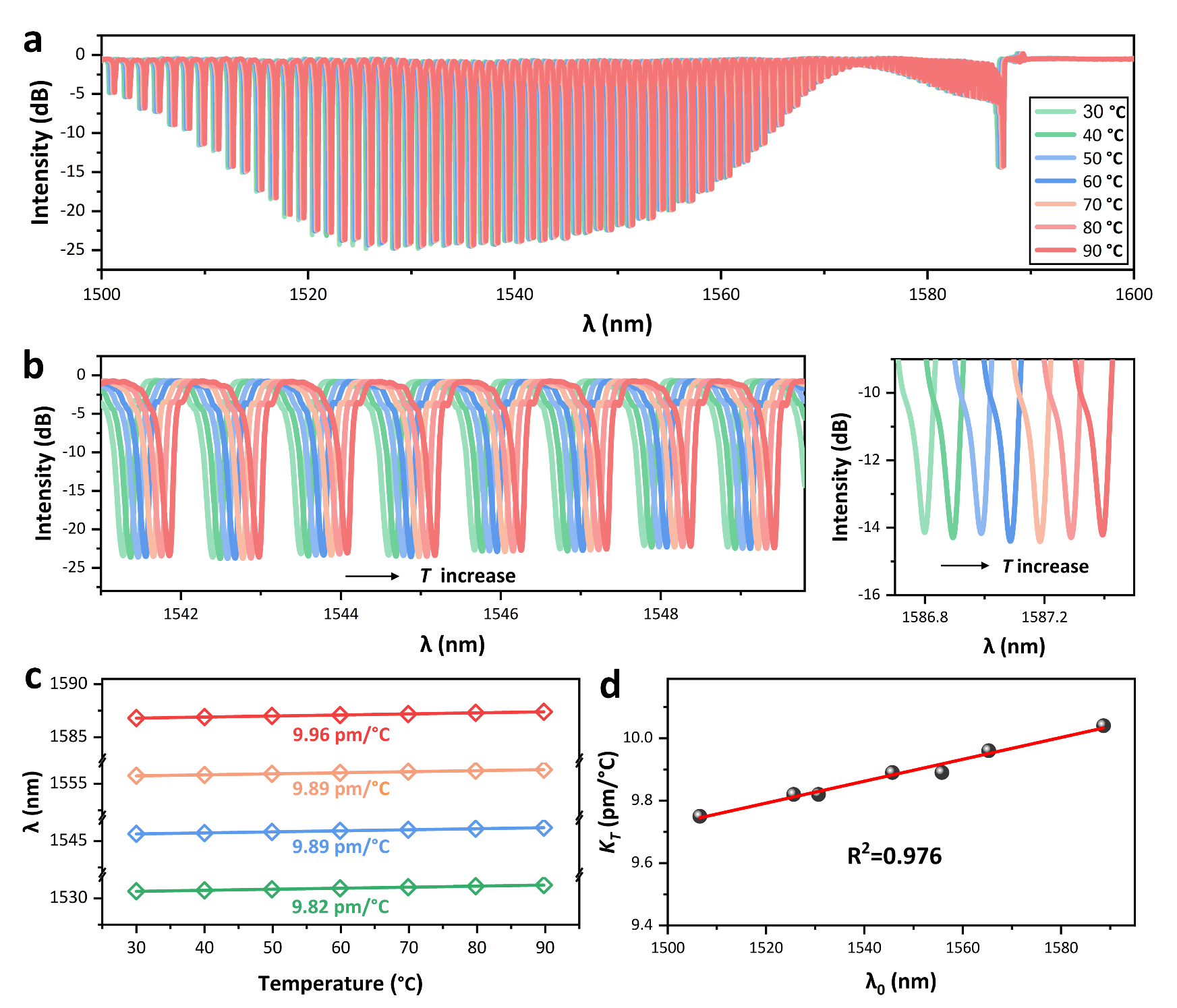


**Fig. S13 Response of the TFBG spectrum to temperature.** **a** The full spectral response of a TFBG at 30 to 90 °C in 10 °C increments in air; **b** Enlarged spectrum of the cladding modes at ~1546 nm (left panel) and ghost mode resonance (right panel). The arrows indicate the direction of wavelength change during increase of temperature. **c** The wavelengths of the cladding modes at ~1,530 (green), ~1,545 (blue), and ~1,555 (orange) nm and ghost resonance (~1,587 nm, red) as a function of temperature. Linear fits to the data yield the thermal sensitivity, *K_T_*). **d** *K_T_* as a function of resonance mode wavelength λ_0_ at 30 °C.


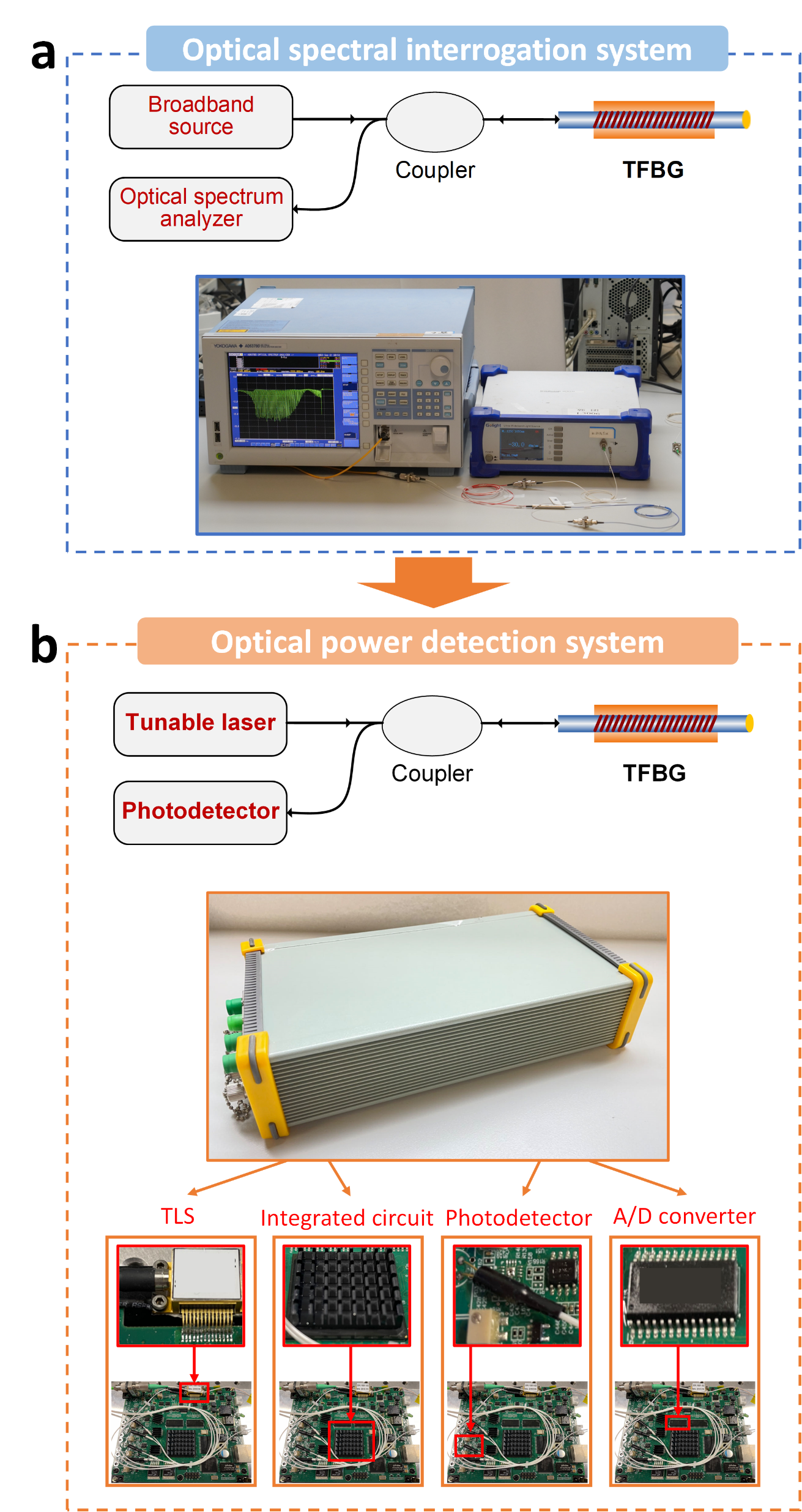


**Fig. S14** **a** Present lab-based spectral interrogation system and **b** the portable power detection instrumentation for in-field measurement.

To realize a portable instrumentation for in-field measurement, we can use a real-time interrogation scheme by replacing the wavelength interrogation by optical power detection. In this case, a tunable laser (TLS) can be used as a source instead of BBS, together with a photodiode as detector and an analog-to-digital converter to obtain the desired data (to replace the OSA, as shown in **Fig. S14a**). The function of the TLS is matching the wavelength of the most sensitive Bragg and Fabry-Perot interference modes, so once that the sensor is characterized it can be replaced by a common laser (for example, a compact VCSEL). This technique relies on the principle of edge filtering so that the optical power change is produced due to the wavelength shift of the mode with respect to the fixed wavelength of the laser source. Our lab made a prototype of the instrumentation using above power detection principle, see the **Fig. S14b**. Such compact interrogator has been reported in several groups.


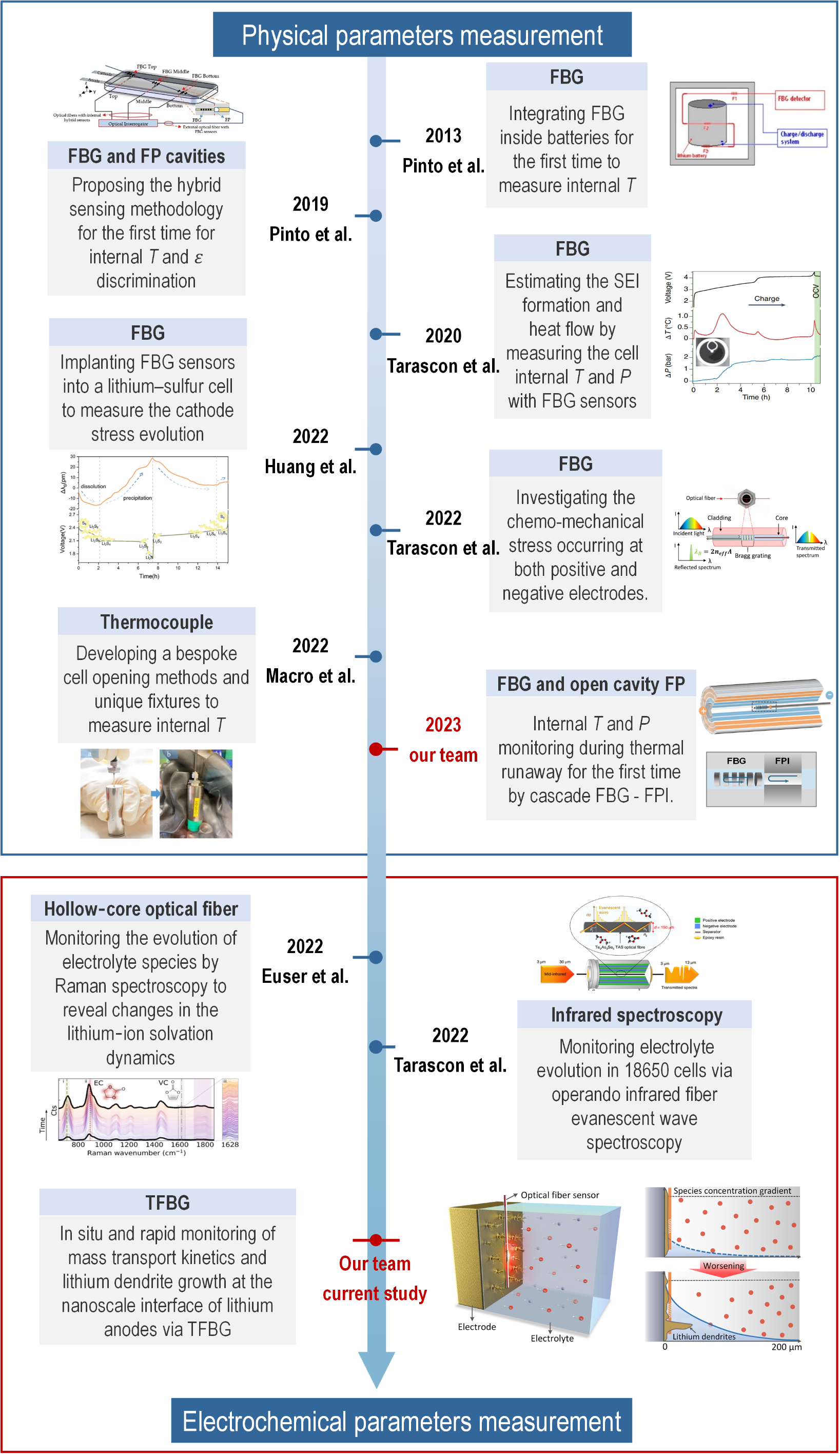


**Fig. S15** A timeline involving the representative references towards internal temperature, strain and electrolyte evolution monitoring of lithium-ion battery.

**Reference**

[1]. Z. X. Huang, N. Yang, J. W. Xie, X. L. Han, X. Y. Yan, D. T. You, K. W. Li, M. I. Vai, K. W. Tam, T. Guo, Improving accuracy and sensitivity of a tilted fiber Bragg grating refractometer using cladding mode envelope derivative, J. Light. Technol. **41**, 4123-4129 (2023).
